# Quantitative Structure–Activity Relationship Evaluation of MDA-MB-231 Cell Anti-Proliferative Leads

**DOI:** 10.3390/molecules26164795

**Published:** 2021-08-07

**Authors:** Ajaykumar Gandhi, Vijay Masand, Magdi E. A. Zaki, Sami A. Al-Hussain, Anis Ben Ghorbal, Archana Chapolikar

**Affiliations:** 1 Department of Chemistry, Government College of Arts and Science, Aurangabad 431 004, Maharashtra, India; dadcguide@gmail.com; 2 Department of Chemistry, Vidya Bharati Mahavidyalaya, Amravati 444 602, Maharashtra, India; vijaymasand@gmail.com; 3 Department of Chemistry, Faculty of Science, Imam Mohammad Ibn Saud Islamic University, Riyadh 13318, Saudi Arabia; sahussain@imamu.edu.sa; 4 Department of Mathematics and Statistics, College of Sciences, Imam Mohammad Ibn Saud Islamic University, Riyadh 13318, Saudi Arabia; assghorbal@imamu.edu.sa

**Keywords:** QSAR, TNBC, MD-MBA-231

## Abstract

In the present endeavor, for the dataset of 219 in vitro MDA-MB-231 TNBC cell antagonists, a (QSAR) quantitative structure–activity relationships model has been carried out. The quantitative and explicative assessments were performed to identify inconspicuous yet pre-eminent structural features that govern the anti-tumor activity of these compounds. GA-MLR (genetic algorithm multi-linear regression) methodology was employed to build statistically robust and highly predictive multiple QSAR models, abiding by the OECD guidelines. Thoroughly validated QSAR models attained values for various statistical parameters well above the threshold values (i.e., R^2^ = 0.79, Q^2^_LOO_ = 0.77, Q^2^_LMO_ = 0.76–0.77, Q^2^-F^n^ = 0.72–0.76). Both de novo QSAR models have a sound balance of descriptive and statistical approaches. Decidedly, these QSAR models are serviceable in the development of MDA-MB-231 TNBC cell antagonists.

## 1. Introduction

Cancer is among the most clinically challenging and life-threatening ailments, globally. In 2020, more than 19.29 million new cancer cases and nearly 10 million related deaths worldwide were chronicled, of which 2.3 million are breast cancer cases with 685,000 related deaths [1,2]. In 2021, female breast cancer has overcome lung cancer and become the most common cancer in the world [2]. Oncology experts prognosticate an estimated more than 16 million breast cancer-induced deaths by 2040. Ample research is being done and researchers are persistently improvising by studying novel treatments and drugs, along with new combinations of existing treatments. Based on the response to various methods of treatment, breast cancer is categorized in three clinical subtypes. Two of them, viz. Hormone Receptor (HR)-positive and Human Epidermal growth factor Receptor 2 (HER2)-positive, are reparative through hormone therapy with or without chemotherapy. In these two subtypes, cancer growth is triggered as a response to the hormones, i.e., estrogen or progesterone, or both receptor (ER/PR) and overexpressed HER2 protein [3,4].

Triple-negative breast cancer (TNBC), the third subtype, unlike the first two, does not contain ER, PR or overexpressed HER2 protein, and this makes it hardest to treat. Therefore, chemotherapy is the mainstay for the treatment of TNBC, essentially at all the stages of breast cancer. Breast oncology is based on in vivo and in vitro studies performed against breast cancer cell lines (BCCL). BT–20 is the first BCCL long-established back in 1958. Despite of tireless work in this area, permanent BCCLs obtained have been notably low in number (about 100 only). Most of the available BCCLs are issued from metastatic tumors, mainly from pleural effusions. MCF-7, T-47D, SK-BR-3 and MDA-MB-231 are a few BCCLs well-reported in breast oncology [5,6].

MDA-MB-231, an epithelial human BCCL, is known to be the most aggressive, invasive and ill differentiate TNBC cell line. Proteolytic degradation of the extracellular matrix brings about invasiveness of the MDA-MB-231 cells [3]. Various genres of compounds such as withangulatin–A derivatives [7], urea-based FGFR1 inhibitors [8], Fam20c inhibitors [9], bradykinin B2 agonists [10], spiroketospirazoles [11], triazole spirodienones [12], calix[4]arene-based carbonyl amide derivatives [13], 6-(2-amino-1H-benzo[d] imidazole-6-yl)quinazolin-4(3H)-one derivatives [14], Purine/purine isostere-based scaffolds as new derivatives of benzamides [15], 5-flurouracil scaffold derivatives [16], pyrimidine-thioindole conjugates [17], thieno[3,2-d]pyrimidine derivatives [18], nitrogen-based chalcogens [19], pyrimidine based benzothiazoles [20], 1,3,4-thiadiazoles [21] and 3-Methylthiazolo[3,2-a]benzimidazole-benzenesulfonamides [22] are tested for their anti-proliferative activities against MDA-MB-231 TNBC cells. Still, the thirst for better anti-TNBC drug candidates is not quenched, and researchers are tantalized for further optimization of the leads.

In silico lead optimization is a feasible, economical, absolutely eco-friendly, quantitatively predictive, relatively faster drug discovery approach, which is the utmost need of time. Sparse animal trials and result accustomization make computer-assisted drug designing (CADD) a pragmatic approach. QSAR is one of the prospering branches of CADD, which persistently and outstandingly contributes towards lead optimization [23,24].

QSAR–a cross-curricular, blended approach, ascertains mathematical correlation between structural traits of the molecule and associated bioactivity on a statistical basis. General steps in a QSAR analysis protocol are (I) selection of a sufficiently large, pertinent molecular dataset with desired bioactivity, (II) 3D structure generation and optimization, (III) molecular descriptor calculation and consequential pruning using an apt statistical method, (IV) QSAR model generation using an algorithm that furnishes propitious molecular descriptors and (V) cromulent validation of developed QSAR model(s) [25]. Descriptive QSAR analysis quantitatively interprets the interrelation of salient but superficially enigmatic molecular structural traits with their reported bioactivity. Statistical QSAR predicts the bioactivity of the molecule prior to its laboratory synthesis and in vivo testing. Coherently balanced descriptive and statistical QSAR improves insight for the pharmacokinetics [25,26,27,28,29,30,31,32]. This underlines the importance of QSAR analysis for further optimization of the leads.

In the present endeavour, a qualitative cum quantitative SAR model for a series of 219 MDA-MB-231 cell anti-proliferative compounds has been performed. The results are useful to optimize compounds for better anti-TNBC activity.

## 2. Results

Although the present study is based on the moderate size dataset, the presence of diverse molecular scaffolds, functional groups, substituents, different rings, viz. non-aromatic, homoaromatic, heteroaromatic, fused rings, spiro compounds, etc., have notably covered an enormous chemical space. Hence, both of the QSAR models generated are based on the divided dataset only.

**Figure 1:**
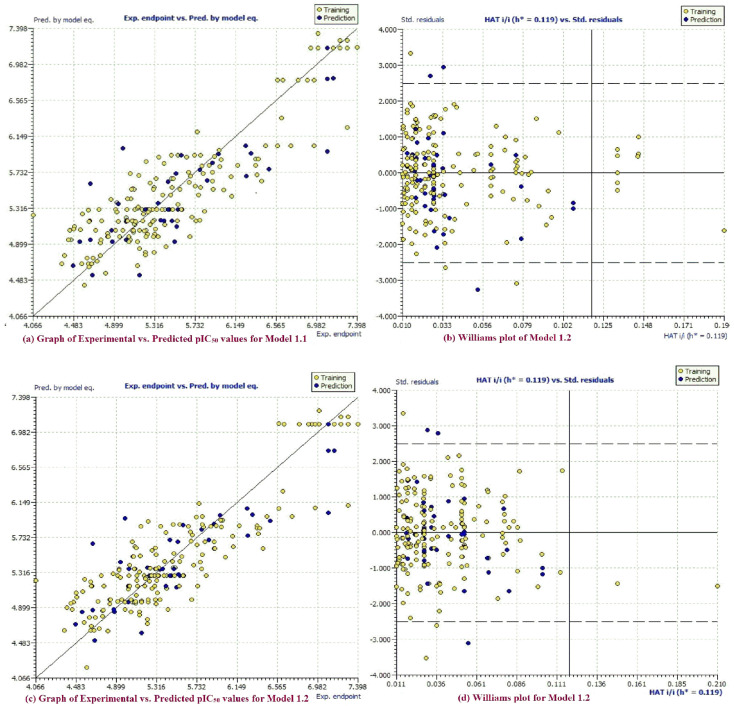
(**a**) Graph of experimental vs. predicted pIC_50_ values for model 1.1; (**b**) Williams plot for model 1.1; (**c**) graph of experimental vs. predicted pIC_50_ values for model 1.2; (**d**) Williams plot for model 1.2.

Fitting parameters, such as R^2^, R^2^_adj_, CCC_tr_, etc., have values well above the approved threshold values, which confirms that the QSAR models are statistically acceptable with an adequate number of molecular descriptors in them. Internal validation parameters such as Q^2^_LOO_, Q^2^_LMO,_ etc. have values that vouchsafe the statistical robustness of the QSAR models (Figure 1a,c). External predictability of both the models is evident from high values of the external validation parameters R^2^_ext_, Q^2^-F^n^, etc. Williams plots for models 1.1 and 1.2 (Figure 1b,d) corroborate the model applicability domain (AD). Accomplishment of approved threshold values for many parameters, as well as low correlation among the molecular descriptors, rules out the possibility of accidental development of the QSAR models [31,32,33,34,35] (Tables S1 and S2; Figures S1a and S2a in supplementary information). These evidences substantiate statistical robustness and good external predictability of these models. 

### 2.1. GA-MLR QSAR Models

#### 2.1.1. Model-1.1 (Divided Set: Training Set–80% and Prediction Set–20%)

pIC_50_ = 4.876(±0.138) + 0.013(±0.006) * **all_MSA3** – 0.538(±0.198) * **com_Splus_7A** + 0.379(±0.265) * **fHringC4B** + 0.107(±0.027) * **fOH5B** – 0.178(±0.077) * **fringNC8B** + 0.924(±0.140) * **com_sp2N_2A**.

R^2^ = 0.79, R^2^_adj_ = 0.78, Q^2^_LOO_ = 0.77, Q^2^_LMO_ = 0.77, RMSEtr = 0.35, MAE_tr_ = 0.27, RSS_tr_ = 21.23, CCC_tr_ = 0.88, RMSE_cv_ = 0.36 MAEcv = 0.28, PRESS_cv_ = 22.89, CCC_cv_ = 0.87, R^2^_ext_ = 0.72, Q^2^-F^1^ = 0.72, Q^2^-F^2^ = 0.72, Q^2^-F^3^ = 0.72.

#### 2.1.2. Model-1.2 (Divided Set: Training Set–80% and Prediction Set–20%)

pIC_50_ = 5.072(±0.193) + 0.012(±0.006) * **all_MSA3** – 0.480(±0.203) * **com_Splus_7A** – 0.096(±0.068) * **com_don_6A** + 0.101(±0.028) * **fOH5B** – 0.160(±0.078) * **fringNC8B** + 0.969(±0.121) * **com_sp2N_2A**.

R^2^ = 0.79, R^2^_adj_ = 0.78, Q^2^_LOO_ = 0.77, Q^2^_LMO_ = 0.76, RMSEtr = 0.35, MAE_tr_ = 0.27, RSS_tr_ = 21.24, CCC_tr_ = 0.88, RMSE_cv_ = 0.36 MAEcv = 0.28, PRESS_cv_ = 22.94, CCC_cv_ = 0.87, R^2^_ext_ = 0.76, Q^2^-F^1^ = 0.76, Q^2^-F^2^ = 0.75, Q^2^-F^3^ = 0.75.

Although for both these models, values of almost all the statistical parameters related to fitting criteria and internal validation are essentially the same, the differences in values of statistical parameters related to external validation, i.e., R^2^_ext_ and Q^2^-F^n^ are noteworthy. This highlights the importance of both of the models. Moreover, these two QSAR models differ in one variable (molecular descriptor) only, viz. **fHringC4B** in model 1.1 (with positive sign of the coefficient) and **com_don_6A** (with negative sign of the coefficient) in model 1.2. In the discussion section, we have explained the importance of these two molecular descriptors, and consequently, the importance of these two QSAR models in terms of their usability and applicability.

## 3. Discussion

Among the reported QSAR studies of MDA-MB-231 TNBC cells, the particularly recent work has been done using the dataset of 61 parthenolide derivatives (*R*^2^ = 0.67, *Q*^2^ = 0.55 and *R*^2^_pred_ = 0.53) [36], and yet another using the dataset of 18β –glycyrrhetinic acid derivatives (*R*^2^ = 0.84, *Q*^2^_LOO_ = 0.83 and *R*^2^_pred_ = 0.75) [37]. These QSAR models are developed on the dataset of compounds with a single scaffold, e.g., parthenolide scaffold, 18β–glycyrrhetinic acid etc., and limited pharmacophoric features. This limits the applicability of these QSAR models. The QSAR models developed in the present work are based on the dataset of relatively large number of compounds with various different scaffolds and large number of pharmacophoric features that have increased the scope of applicability of these models. Subjective feature selection provided some simple molecular descriptors; those are reflected in the QSAR models. Values of these molecular descriptors can be easily modified by introducing some simple constitutional and structural alterations to bring about optimization.

Both models 1.1 and 1.2 have been constructed using the divided dataset only. These models differ in only one descriptor out of a total of six descriptors. Although, the change in the activity of each molecule, to a large extent, is a combined effect of all the six molecular descriptors, in the ensuing section effect of variation in each molecular descriptor on biological activity of the irrespective molecule, and is illustrated with examples (see supplementary information Table S3).

**1.**  **all_MSA3, fHringC4B, fOH5B and com_sp2N_2A:** All of these molecular descriptors have positive values of the coefficient and increase in the values of these molecular descriptors, which increases MDA-MB-231 anti-proliferative activity.

**Figure 2:**
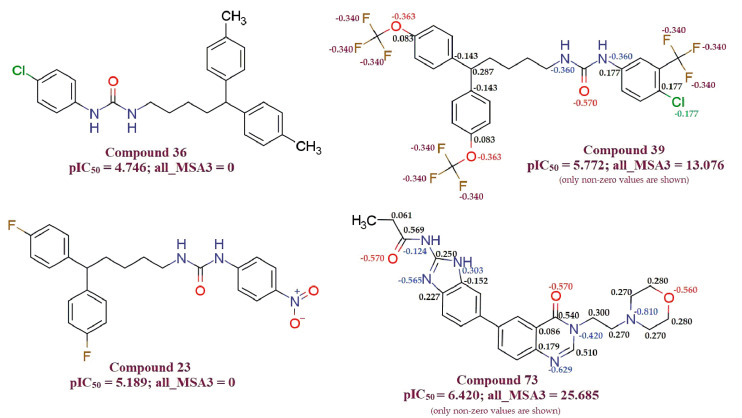
Illustration for molecular descriptor **all_MSA3**.

**all_MSA3** (Molecular Surface Area of all atoms having partial charge in the range of 0.099 to 0.000): The observation is supported by comparing compound 36 (IC_50_ = 4.746) with compound 39 (pIC_50_ = 5.772), for which an increase in the value of **all_MSA3** from 0 for compound 36 to 13.07 for compound 39 results in an increase in pIC_50_ value by about 1 unit (about ten-fold increase in MDA-MB-231 cell anti-proliferative activity). Compound 23 (**all_MSA3** = 0; pIC_50_ = 5.189) and compound 73 (**all_MSA3** = 25.69; pIC_50_ = 6.420) is another pair used as an example to support this observation. Partial charges on each atom of the compound with the non-zero value of the **all_MSA3** descriptor are shown explicitly in Figure 2. The descriptor **all_MSA3** to calculate molecular surface area takes into consideration atoms with partial charges in the range −0.099 to 0.000 only.

**Figure 3:**
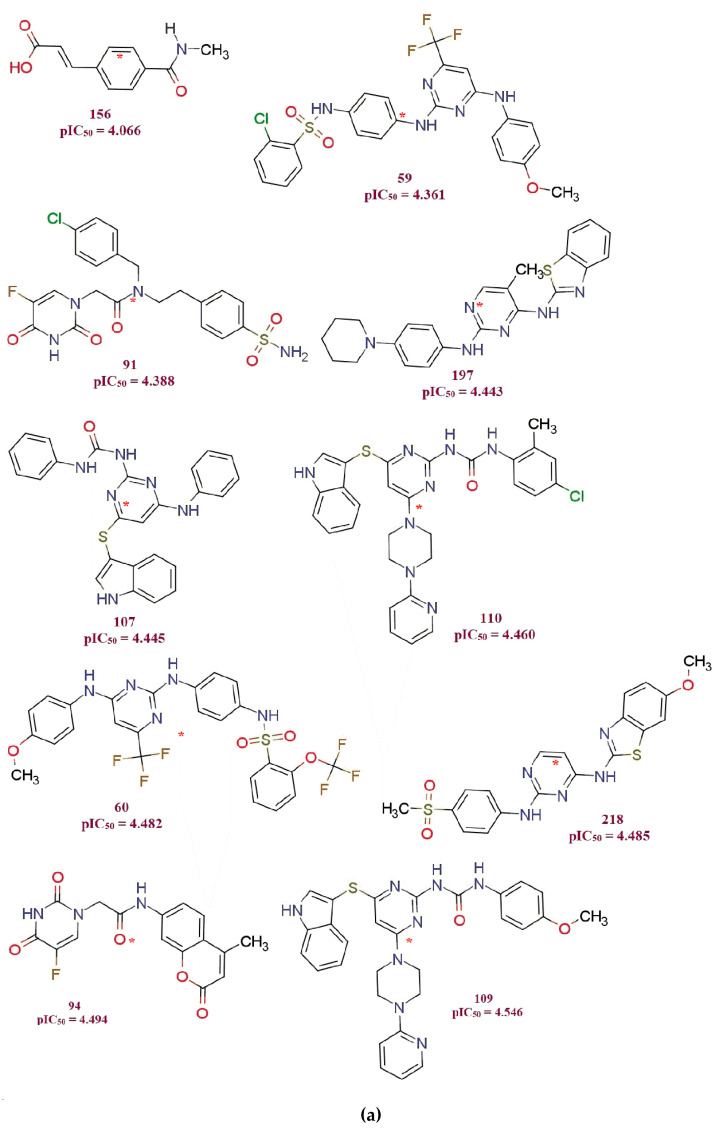

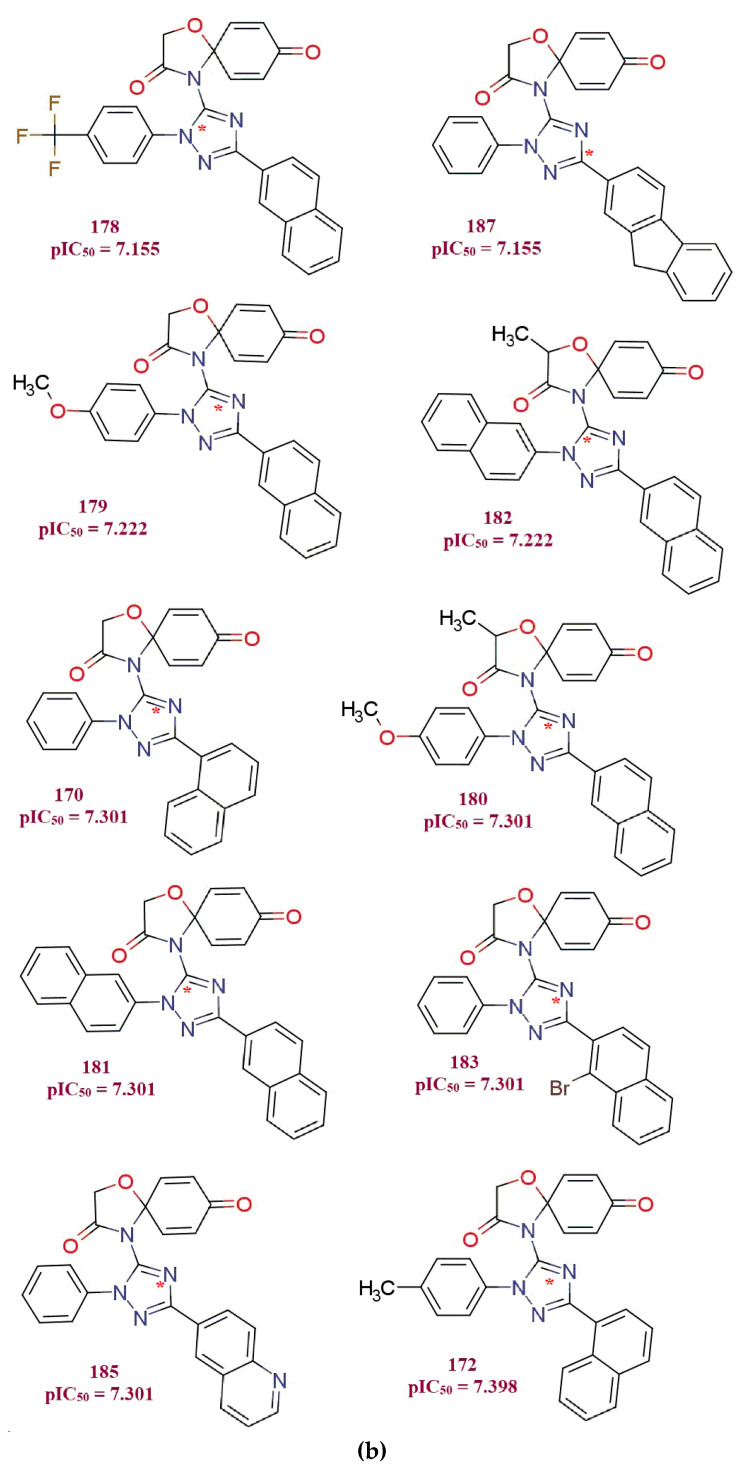
Variations in activity and chemical structures in the present dataset of MDA-MB-231 anti-prolifertives: (**a**) ten least active compounds; (**b**) ten most active compounds from the present series. Asterisks * mark denotes the position of the center of the mass of the molecules.

**Figure 4:**
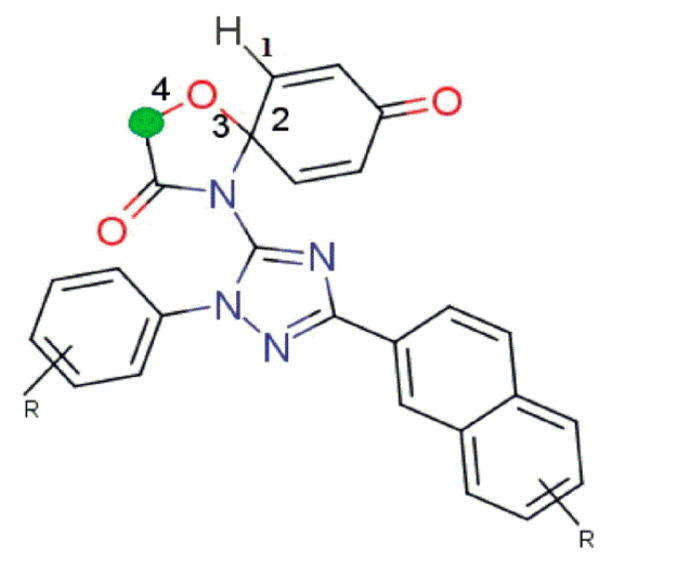
Illustration for molecular descriptor **fHringC4B**.

**fHringC4B** (Frequency of occurrence of ring carbons which are present exactly at four bonds from the hydrogen atom): this molecular feature is absent in all the ten least active compounds but present in all the ten most active compounds (see Figure 3a,b and Figure 4), which highlights the importance of the presence of this molecular feature for better MDA-B-231 anti-proliferative activity in the compound.

**Figure 5:**
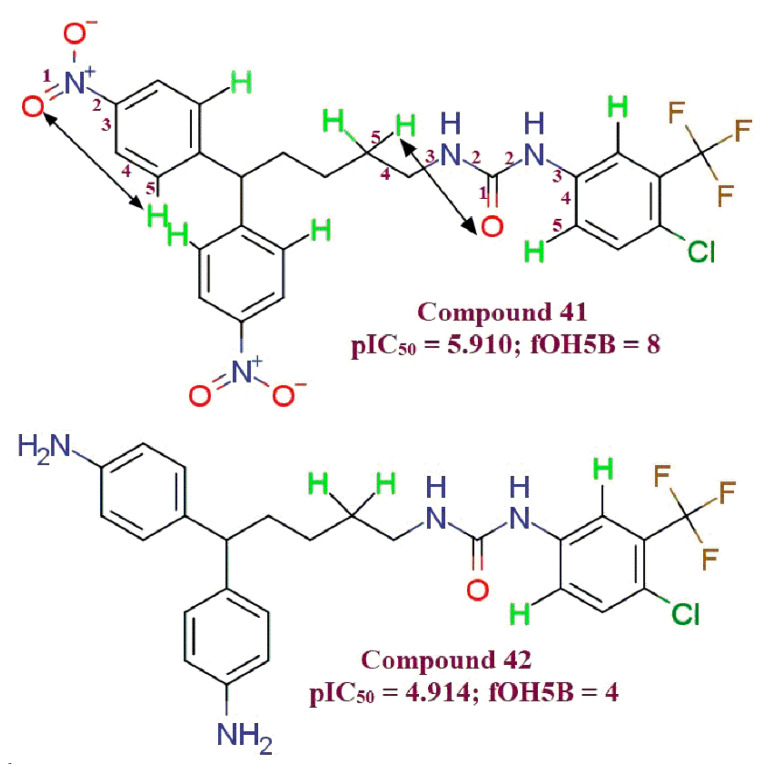
Illustration for molecular descriptor **fOH5B**. (Distinguished hydrogens are shown in green color).

**fOH5B** (Frequency of occurrence of number of hydrogen atoms, which are present exactly at 5 bonds from Oxygen atom): In compound 42 (**fOH5B** = 4; pIC_50_ = 4.914) there are four hydrogen atoms which are five bonds away from oxygen. Whereas, in ten-fold more potent MDA-MB-231 anti-proliferative compound 41 (**fOH5B** = 8, pIC_50_ = 5.910), eight such hydrogens are present. This illustrates the significance of the higher value of the **fOH5B** molecular descriptor in order for leads to be better MBA-MD-231 anti-proliferative agents (Figure 5).

**com_sp2N_2A** (Number of sp2-nitrogen atoms within 2Å from center of mass of molecule): Significance of this molecular descriptor can be rationalized with the fact that in all the ten least active compounds (Figure 3a), this descriptor gets a value of zero, and in all the ten most active compounds, the value of this descriptor is two (except for compound 183, which has one sp2-N atom within 2Å from the center of the mass of the molecule). (Center of mass in each molecule from Figure 3a shown with red asterisk ‘*’ mark)

**2.**  **com_Splus_7A, com_don_6A and fringNC8B**: These three molecular descriptors have negative coefficients and hence decrease in their values possibly will increase MDA-MB-231 anti-proliferative activity.

**com_Splus_7A** (Number of positively charged Sulfur atoms within 7Å from the center of the mass of the molecule) There is no positively charged sulfur atom within 7Å from the center of the mass of molecule (**com_Splus_7A** = 0), rather the sulfur atom itself is absent in these ten most active compounds (pIC_50_ = 7.155–7.398) (Figure 3b). All the compounds having a unit of pIC_50_ > 5.223 have the **com_Splus_7A** descriptor value as zero (Table S3 in supplementary information). (Center of mass in each molecule in Figure 3b shown with red asterisk ‘*’ mark)

**com_don_6A** (Number of donor atoms within 6Å from the center of the mass of the molecule): There is no donor atom within 6Å from the center of the mass of the molecule (**com_don_6A**=0); in fact, there is no H-Donor functionality such as N-H, O-H present in all the ten most active compounds (pIC_50_ = 7.155–7.398). Hence, there is great scope to say that the zero value for the **com_Splus_7A** and **com_don_6A** molecular descriptors have gained better MDA-MB-231 cell anti-proliferation potency to them. With two H-donor atoms (in functionality –COOH and –NH-), Compound 156 (**com_don_6A** = 2; pIC_50_ = 4.066) is the least active compound of the series that highlights the importance of absence of **com_don_6A** molecular descriptor for molecule to be better MDA-MB-231 cell anti-proliferator. Comparison of compound 97 (**com_don_6A** = 3; pIC_50_ = 5.159) with 148 (**com_don_6A** = 0; pIC_50_ = 5.212) and 95 (**com_don_6A** = 3; pIC_50_ = 4.916) with 107(**com_don_6A** = 4; pIC_50_ = 4.445) also support the observation.

**Figure 6:**
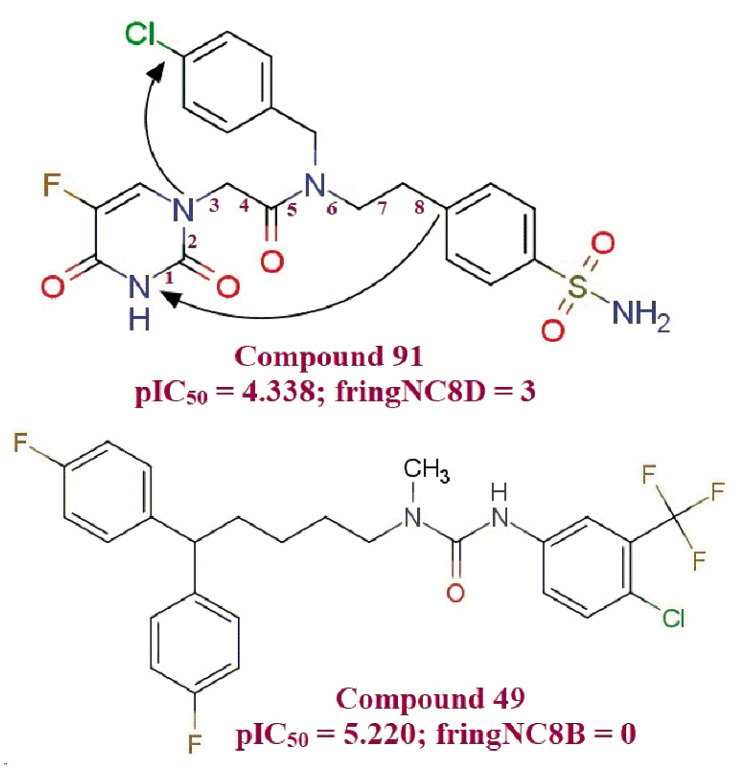
Illustration for molecular descriptor **fringNC8B**.

**fringNC8B** (Frequency of occurrence of number of carbon atoms which are present exactly at 8 bonds from ring nitrogen atoms): Compound 49 (IC_50_ = 6.02 μM) with no such carbon atoms which are present exactly at eight bonds from the ring nitrogen atom found to be about eight-fold more potent than compound 91 (IC_50_ = 40.92 μM), which contain three such carbons (Figure 6). Moreover, all the ten most active compounds of the series show an absence of carbon atoms, which are present eight bonds away from ring nitrogen (**fringNC8B** = 0). These observations mark the importance of the **fringNC8B** molecular descriptor.

Two QSAR models proposed in the present study differ in one variable (molecular descriptor) only, viz. **fHringC4B** in model 1.1 (with positive coefficient) and **com_don_6A** (with negative coefficient) in model 1.2. All the relatively potent MDA-MB-231 cell anti-proliferative compounds from the present dataset with pIC_50_ ≥ 7 have **fHringC4B = 1** and **com_don_6A** = 0 (except compound 154 with **fOH5B** = 9, which more than compensates the counter effects of the rest of the molecular descriptors) and all the relatively inactive compounds with pIC_50_ ≤ 5.043 have **fHringC4B** = 0 and **com_don_6A** > 0 (i.e., non-zero). This observation signifies the importance of combined effect of the molecular features represented with these molecular descriptors, and eventually broadens the applicability of the present QSAR evaluation studies (see supplementary information Table S3).

**Figure 7:**
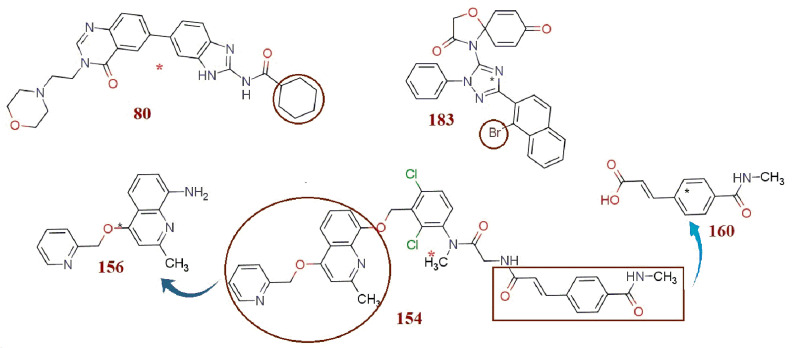
Representation and illustration of outliers.

There are five outliers, viz. molecules 80, 154, 156, 160 and 183 (with >2.5 σ), which are revealed in the Williams plot (see Figure 1b–d, Figure 7 and supplementary information Tables S1–S3, Figures S1a and S2a). Lipophilic cyclohexyl substituent in compound 80 (Practical pIC_50_ = 4.991, Predicted pIC_50_ = 6.015 by Model 1.1 and Predicted pIC_50_ = 5.961 by Model 1.2) leads to the increase in values of **all_MSA3** and **fOH5B** molecular descriptors due to an increase in –CH_2_- groups in the molecule. Hence, both the models predicted extremely high pIC_50_ values for molecule 80. Experimentally, the typical non-planar, chair conformation of the cyclohexyl group due to steric reasons might have restricted inhibitory interaction of compound 80 with the target (pIC_50_ = 4.991), and hence compound 80 turned out as an outlier. Compound 154 (Practical pIC_50_ = 7.097, Predicted pIC_50_ = 5.977 by Model 1.1 and Predicted pIC_50_ = 6.031 by Model 1.2) consists of multiple scaffolds with different pharmacophores which (pIC_50_ = 7.097) might practically have increased the possible inhibitory potency of the molecule through an increased number of favorable interactions, but owing to the same reason, there is an increase in the value of **fringNC8B** and **com_don_6A** molecular descriptors (both have negative coefficient), and this leads to the extremely low pIC_50_ value prediction by both of the models.

Compound 156 (Practical pIC_50_ = 4.066, Predicted pIC_50_ = 5.239 by Model 1.1, Predicted pIC_50_ = 5.227 by Model 1.2) and compound 160 (Practical pIC_50_ = 4.658, Predicted pIC_50_ = 5.604 by Model 1.1 and Predicted pIC_50_ = 5.664 by Model 1.2) are developed from some of the structural fragments of compound 154 (Figure 7). These fragments retained their molecular features, which helped these compounds to attain enough large values for a few molecular descriptors, with which both the models predicted high pIC_50_ values for these molecules, but practically, these molecules fall short in a few other molecular features such as length of the molecule, which limited their inhibitory potency and set them in the outliers’ category.

The **com_sp2N_2A** molecular descriptor does not take into consideration any such sp2-nitrogen, which is beyond 2Å from the center of the mass of the molecule. In compound 183, (Practical pIC_50_ = 7.301, Predicted pIC_50_ = 6.253 by Model 1.1, Predicted pIC_50_ = 6.113 by Model 1.2), presence of a Bromine (Br)-heavy atom with large atomic/ionic volume as a substituent caused shifting of the position of the center of the mass of the molecule, which reduced the value of **com_sp2N_2A** (positive coefficient) to 1, and hence both models predicted lower pIC_50_ values than was experimentally observed.

## 4. Materials and Methods

### 4.1. Dataset Selection

A total of 219 MDA-MB-231 cell antagonists having moderate anti-proliferation potency (experimental IC_50_ = 0.04 to 86 μM) have been selected for the present work [8,9,10,11,12,14,15,16,17,18,19,20]. The IC_50_ values were converted to pIC_50_ (pIC_50_ = –logIC_50_) before actual QSAR analysis. To demonstrate the variation in bioactivity with chemical features covered by the present dataset, ten least and ten most active molecules have been depicted in Figure 3a,b. The SMILES strings with reported IC_50_ and pIC_50_ values for all the molecules are present in Table S4 in the supplementary material.

### 4.2. Molecular Structure Drawing and Optimization

A free comprehensive chemical drawing package, ChemSketch 12 Freeware (www.acdlabs.com, accessed on 15 May 2021), was used to draw structures of all 219 molecules. Subsequent conversion of these structures to corresponding 3D structures was achieved using another free and open-source chemistry toolbox, OpenBabel 2.4.0. Thereafter, an optimization and molecular alignment was carried out using the force field MMFF94 available in TINKER (default settings) and Open3DAlign, respectively.

### 4.3. Molecular Descriptor Calculation and Molecular Descriptor Pruning

PyDescriptor calculated various molecular descriptors for each molecule [38]. More than 15,000 molecular descriptors had been provided by PyDescriptor for each molecule. It is necessarily important to remove redundant molecular descriptors to steer clear of the impinging of multi-collinear and spurious variables in the GA-MLR model. Hence, molecular descriptors with nearly constant values (>95%) and co-linearity (|R|) >0.95 were removed using objective feature selection (OFS) in QSARINS v2.2.4 [39,40]. The contracted molecular descriptor pool thus resulted is comprised of 1370 molecular descriptors only.

### 4.4. QSAR Model–Development and Validation

The contracted molecular descriptor set is a heterogeneous set of descriptors in the sense that it comprises 0D to 3D descriptors, charge descriptors and molecular properties, etc. that have covered an adequately comprehensive descriptor space. Subjective feature selection (SFS) in QSARINS v2.2.4 was executed in order to construct statistically robust GA-MLR-based QSAR models. Thereafter, thorough statistical validation (internal and external), Y-randomization and applicability domain analysis of the derived models was done, abiding by OECD [41,42,43,44] principles. A dataset of 219 molecules is large enough that even on splitting, the size of training dataset has covered the chemical space adequately and to great extent. Following are the steps in protocol for QSAR model construction using the divided dataset:

**i.**  As per OECD guidelines, thorough internal as well as external validation of the developed QSAR model(s), for example, is necessarily mandatory. Hence, some molecules from the dataset were randomly kept aside as a prediction set, and remaining molecules (training set) were subjected to SFS treatment to develop the QSAR model. The QSAR model(s) generated is validated using molecules in the prediction set.
  Random splitting of the dataset using random splitting option in QSARINS v.2.2.4 into an 80% training set (175 molecules in training set) and a 20% prediction set (44 molecules in prediction set) was achieved. The training set was used for QSAR model development, and the prediction set was utilized for external validation.
**ii.**  QSARINS v2.2.4 with default settings and Q^2^_LOO_ as a fitness function for feature selection was deployed in genesis of the GA-MLR-based QSAR models with double cross validation. Up to six variables, there was a generous increase in the Q^2^_LOO_ value, but minor augmentation was observed thereafter. Consequently, the selection of the molecular descriptor was confined to a set of six descriptors to foil the danger of over-fitting, and this additionally helped to derive easy and informative QSAR models (see supplementary information Table S3 values for all the selected molecular descriptors present in QSAR models).
**iii.**  Abide by OECD guidelines; for ensured proper validation, all the models were subjected to internal and external validation, Y-randomization and model applicability domain (AD) analysis using QSARINS 2.2.4. Robustness of the GA-MLR-based QSAR model was adjudicated on the basis of (a) internal validation based on Leave-One-Out (LOO) and Leave-Many-Out (LMO) procedure; (b) external validation; (c) Y-randomization and (d) fulfilling of the respective threshold value for the statistical parameters: R^2^ ≥ 0.6, Q^2^_LOO_ ≥ 0.5, Q^2^_LMO_ ≥ 0.6, R^2^ > Q^2^, R^2^_ex_ ≥ 0.6, RMSE_tr_ < RMSE_cv_, ΔK ≥ 0.05, CCC ≥ 0.80, Q^2^-F^n^ ≥ 0.60, r^2^m ≥ 0.6, 0.9 ≤ k ≤ 1.1, 0.9 ≤ k’ ≤ 1.1 with RMSE and MAE close to zero. All QSAR models which failed to meet any of these criteria were omitted. Two QSAR models (1.1 and 1.2) with best values of these parameters and with best predicative ability (Q^2^-F^n^ > 0.71) were selected.

## 5. Conclusions

A well-founded balance of statistical QSAR and descriptive QSAR with highly precise bioactivity predictability (external predictability) on incorporation of molecular features is furnished by both models. Various statistical parameters that are indicators of preciseness of the external predictability, especially R^2^_ext_ and Q^2^-F^n^, resulted in extremely high values for both models. Molecular features which appeared in QSAR models, such as an increased number of four bond-distant ring carbons from hydrogen, five bond-distant hydrogen from oxygen, a lesser number of eight-bond distant carbons from ring nitrogen, etc., are easy to incorporate in a manner that will make optimization easy and scopeful. These models will help in optimizing present compounds to more potent MBA-MD-231 anti-proliferative leads to treat triple-negative breast cancer.

## Data Availability

The data are available in the supplementary section.

## Supplementary Materials

The following are available online at https://www.mdpi.com/article/10.3390/molecules26164795/s1, Table S1: Details regarding performance of model 1.1. Table S2: Details regarding performance of model 1.2. Table S3: The values for selected molecular descriptors present in QSAR models. Table S4: The SMILES notation for two hundred and nineteen MDA-MB-231 cell anti-proliferative leads, along with their reported IC_50_ and pIC_50_ values; Figure S1: Different graphs associated with model 1.1 (a) graph of pred. endpoint vs. residual values (b) Y-scrambling plot. Figure S2: Different graphs associated with model 1.2 (a) graph of pred. endpoint vs. residual values (b) Y-scrambling plot. Statistical parameters for used for validation of QSAR models

## Abbreviations

**CADD**	Computer-Aided Drug Designing
**SMILES**	Simplified Molecular-Input Line-Entry System
**GA**	Genetic Algorithm
**MLR**	Multiple Linear Regression
**QSAR**	Quantitative Structure–Activity Relationship
**OLS**	Ordinary Least Square
**QSARINS**	QSAR Insubria
**OECD**	Organization for Economic Co-operation and Development
**OFS**	Objective Feature Selection
**SFS**	Subjective Feature Selection
**HER2**	Human Epidermal growth factor Receptor 2
**TNBC**	Triple Negative Breast Cancer
**ER**	Estrogen Receptor
**PR**	Progesterone Receptor
**BCCL**	Breast Cancer Cell Line
**LOF**	Lack of Fit (Friedmann Parameter)
**RMSE**	Root Mean Square Error
**MAE**	Mean Absolute Error
**RSS**	Residual Sum of Squares
**CCC**	Concordance Correlation Coefficient
**PRESS**	Predictive Residual Sum of Squares
**LOO**	Leave One Out
**LMO**	Leave Many Out
