# Supplementary material for: Quantitative Structure–Activity Relationship Evaluation of MDA-MB-231 Cell Anti-Proliferative Leads
Source: Molecules. 2021 Aug 7;26(16):4795. doi: 10.3390/molecules26164795 (PMC8401583; doi:10.3390/molecules26164795)
Supplement: Supplementary file 1 [file molecules-26-04795-s001.zip › molecules-1280365-supplementary.pdf]

Quantitative Structure Activity Relationship Evaluation of MDA-MB-231 cell Anti-Proliferative Leads

➢ **Supplementary Information:**

Table S1: Details regarding performance of model 1.1.

ID	Status	Exp. endpoint	Pred. by model eq.	Pred.Mod. Eq.Res.	Pred. LOO	Pred. LOO Res.	HAT i/i (h*=0.1186)	Std.Pred. Mod.Eq. Res.	Std.Pred. LOO Res.
1	Training	5.664	5.7385	0.0745	5.7399	0.0759	0.0182	0.2129	0.2168
2	Training	6	5.8829	-0.1171	5.8775	-0.1225	0.0443	-0.3389	-0.3546
3	Training	6.174	5.8145	-0.3595	5.8036	-0.3704	0.0294	-1.0326	-1.0639
4	Prediction	6.31	5.9553	-0.3547	-	-	0.027	-1.0177	-1.0177
5	Prediction	5.971	5.9475	-0.0235	-	-	0.0264	-0.0673	-0.0673
6	Training	6.357	5.8162	-0.5408	5.7996	-0.5574	0.0297	-1.5537	-1.6013
7	Prediction	6.495	5.7691	-0.7259	-	-	0.0303	-2.0861	-2.0861
8	Training	5.093	5.5462	0.4532	5.5548	0.4618	0.0186	1.2945	1.319
9	Training	5.686	5.6905	0.0045	5.6908	0.0048	0.0434	0.0132	0.0138
10	Prediction	5.456	5.628	0.172	-	-	0.0302	0.4942	0.4942
11	Training	5.728	5.9309	0.2029	5.9362	0.2082	0.0254	0.5815	0.5967
12	Training	5.79	5.9324	0.1424	5.9361	0.1461	0.0255	0.4082	0.4189
13	Training	5.991	5.7995	-0.1915	5.7943	-0.1967	0.0268	-0.5492	-0.5643
14	Prediction	5.78	5.765	-0.015	-	-	0.03	-0.0432	-0.0432
15	Training	5.073	5.4257	0.3527	5.4305	0.3575	0.0133	1.0049	1.0185
16	Training	5.738	5.5715	-0.1665	5.5679	-0.1701	0.0215	-0.4762	-0.4866
17	Training	5.879	5.8195	-0.0595	5.818	-0.061	0.0253	-0.1705	-0.1749
18	Training	5.807	5.8093	0.0023	5.8093	0.0023	0.0258	0.0065	0.0066
19	Training	6.102	5.6871	-0.4149	5.6813	-0.4207	0.0138	-1.1822	-1.1987
20	Prediction	5.86	5.6377	-0.2223	-	-	0.0287	-0.6383	-0.6383
21	Training	5.115	5.3052	0.1902	5.3108	0.1958	0.0283	0.5461	0.5621
22	Training	4.928	5.3833	0.4553	5.391	0.463	0.0166	1.2993	1.3212
23	Training	5.189	5.3052	0.1162	5.3086	0.1196	0.0283	0.3337	0.3434
24	Training	5.471	5.3052	-0.1658	5.3004	-0.1706	0.0283	-0.4759	-0.4897
25	Prediction	5.556	5.3052	-0.2508	-	-	0.0283	-0.7199	-0.7199
26	Training	5.295	5.3052	0.0102	5.3055	0.0105	0.0283	0.0294	0.0302
27	Training	5.421	5.3052	-0.1158	5.3019	-0.1191	0.0283	-0.3323	-0.342
28	Training	5.502	5.3052	-0.1968	5.2995	-0.2025	0.0283	-0.5649	-0.5813
29	Training	5.391	5.3052	-0.0858	5.3027	-0.0883	0.0283	-0.2462	-0.2534
30	Training	5.034	5.3052	0.2712	5.3131	0.2791	0.0283	0.7787	0.8014
31	Training	5.04	5.4304	0.3904	5.4353	0.3953	0.0124	1.1118	1.1258
32	Training	5.46	5.3052	-0.1548	5.3007	-0.1593	0.0283	-0.4443	-0.4572
33	Prediction	5.342	5.3052	-0.0368	-	-	0.0283	-0.1055	-0.1055
34	Prediction	5.354	5.3773	0.0233	-	-	0.0173	0.0666	0.0666
35	Training	5.6	5.3052	-0.2948	5.2966	-0.3034	0.0283	-0.8462	-0.8709
36	Training	4.746	5.3052	0.5592	5.3215	0.5755	0.0283	1.6055	1.6523
37	Training	5.481	5.4734	-0.0076	5.4733	-0.0077	0.0105	-0.0217	-0.0219
38	Training	5.399	5.4736	0.0746	5.4744	0.0754	0.0105	0.2122	0.2145
39	Training	5.772	5.4703	-0.3017	5.4671	-0.3049	0.0105	-0.8582	-0.8674

40	Prediction	5.243	5.3052	0.0622	-	-	0.0283	0.1787	0.1787
41	Training	5.91	5.735	-0.175	5.7222	-0.1878	0.0678	-0.513	-0.5503
42	Training	4.914	5.3052	0.3912	5.3166	0.4026	0.0283	1.1232	1.1559
43	Training	5.298	5.3052	0.0072	5.3054	0.0074	0.0283	0.0208	0.0214
44	Training	5.264	5.3052	0.0412	5.3064	0.0424	0.0283	0.1184	0.1218
45	Prediction	5.456	5.3052	-0.1508	-	-	0.0283	-0.4328	-0.4328
46	Training	5.59	5.3052	-0.2848	5.2969	-0.2931	0.0283	-0.8175	-0.8413
47	Training	5.437	4.8755	-0.5615	4.8527	-0.5843	0.039	-1.6209	-1.6868
48	Training	5.339	5.3052	-0.0338	5.3043	-0.0347	0.0283	-0.0969	-0.0997
49	Prediction	5.22	5.3052	0.0852	-	-	0.0283	0.2447	0.2447
50	Prediction	4.942	5.3695	0.4275	-	-	0.0183	1.221	1.221
51	Training	4.931	5.2729	0.3419	5.2785	0.3475	0.0162	0.9754	0.9914
52	Training	5.248	5.2731	0.0251	5.2745	0.0265	0.0515	0.073	0.077
53	Training	4.943	5.1633	0.2203	5.1796	0.2366	0.069	0.646	0.6938
54	Training	5.315	4.8064	-0.5086	4.6837	-0.6313	0.1944	-1.6035	-1.9904
55	Training	4.681	4.743	0.062	4.7482	0.0672	0.0764	0.1827	0.1978
56	Training	4.556	5.0685	0.5125	5.1173	0.5613	0.087	1.5179	1.6625
57	Prediction	4.674	4.5441	-0.1299	-	-	0.0785	-0.3831	-0.3831
58	Training	4.633	4.6728	0.0398	4.6761	0.0431	0.0756	0.1172	0.1268
59	Training	4.361	4.6739	0.3129	4.6994	0.3384	0.0755	0.9208	0.996
60	Prediction	4.482	4.6509	0.1689	-	-	0.0753	0.4969	0.4969
61	Training	4.756	4.7676	0.0116	4.7687	0.0127	0.0843	0.0343	0.0374
62	Training	4.623	4.7443	0.1213	4.7544	0.1314	0.0763	0.3572	0.3868
63	Training	4.814	4.5658	-0.2482	4.5459	-0.2681	0.074	-0.73	-0.7883
64	Prediction	5.163	4.5425	-0.6205	-	-	0.0783	-1.8289	-1.8289
65	Training	4.566	4.6731	0.1071	4.6818	0.1158	0.0755	0.3152	0.3409
66	Training	4.673	4.6735	0.0005	4.6736	0.0006	0.0755	0.0015	0.0016
67	Training	4.762	4.9157	0.1537	4.9292	0.1672	0.0809	0.4536	0.4935
68	Training	5.223	4.8072	-0.4158	4.7631	-0.4599	0.0959	-1.2374	-1.3687
69	Training	4.634	5.2994	0.6654	5.327	0.693	0.0399	1.9217	2.0015
70	Training	5.143	5.3211	0.1781	5.3309	0.1879	0.0524	0.5176	0.5462
71	Training	4.87	5.4192	0.5492	5.4357	0.5657	0.0292	1.5774	1.6249
72	Training	6.027	5.5292	-0.4978	5.5239	-0.5031	0.0106	-1.4162	-1.4314
73	Training	6.42	5.6305	-0.7895	5.6156	-0.8044	0.0185	-2.2551	-2.2976
74	Training	5.91	5.6316	-0.2784	5.6263	-0.2837	0.0187	-0.7952	-0.8103
75	Training	5.415	5.8386	0.4236	5.8474	0.4324	0.0203	1.211	1.2361
76	Prediction	5.914	5.8437	-0.0703	-	-	0.0208	-0.2011	-0.2011
77	Training	5.314	5.5587	0.2447	5.5616	0.2476	0.0119	0.6965	0.7049
78	Training	5.754	6.2035	0.4495	6.2342	0.4802	0.064	1.3148	1.4047
79	Training	5.793	5.5577	-0.2353	5.5549	-0.2381	0.0118	-0.6698	-0.6778
80	Prediction	4.991	6.0152	1.0242	-	-	0.0342	2.9492	2.9492
81	Prediction	6.26	5.6947	-0.5653	-	-	0.0287	-1.6233	-1.6233
82	Training	5.489	5.6629	0.1739	5.6925	0.2035	0.1454	0.5323	0.6228
83	Training	5.333	5.6648	0.3318	5.7213	0.3883	0.1455	1.0157	1.1886
84	Training	5.116	5.1279	0.0119	5.128	0.012	0.0123	0.0338	0.0342
85	Training	5.511	5.6624	0.1514	5.6881	0.1771	0.1454	0.4633	0.5421
86	Training	5.83	5.2847	-0.5453	5.2766	-0.5534	0.0146	-1.5546	-1.5777
87	Training	5.499	5.5614	0.0624	5.5639	0.0649	0.0396	0.1801	0.1875
88	Training	5.274	5.1289	-0.1451	5.1271	-0.1469	0.0122	-0.4131	-0.4182
89	Training	5.423	5.1268	-0.2962	5.1231	-0.2999	0.0123	-0.8434	-0.8539
90	Training	4.594	4.4254	-0.1686	4.408	-0.186	0.0938	-0.5011	-0.553

91	Training	4.388	4.77	0.382	4.8123	0.4243	0.0998	1.1393	1.2656
92	Training	5.164	4.8542	-0.3098	4.8241	-0.3399	0.0887	-0.9183	-1.0077
93	Training	4.641	4.6379	-0.0031	4.6376	-0.0034	0.079	-0.0092	-0.01
94	Training	4.494	5.1101	0.6161	5.1327	0.6387	0.0353	1.7752	1.8402
95	Training	4.916	5.0625	0.1465	5.0645	0.1485	0.0132	0.4175	0.423
96	Training	5.1	5.0395	-0.0605	5.038	-0.062	0.0236	-0.1733	-0.1775
97	Training	5.159	5.0604	-0.0986	5.0591	-0.0999	0.0132	-0.2808	-0.2846
98	Training	5.063	4.9531	-0.1099	4.951	-0.112	0.019	-0.3141	-0.3201
99	Training	5.226	4.7768	-0.4492	4.7578	-0.4682	0.0405	-1.2978	-1.3525
100	Prediction	4.994	5.0613	0.0673	-	-	0.0132	0.1918	0.1918
101	Training	5.139	4.9319	-0.2071	4.9247	-0.2143	0.0339	-0.5962	-0.6171
102	Training	5.09	5.0613	-0.0287	5.0609	-0.0291	0.0132	-0.0818	-0.0829
103	Training	5.103	5.0618	-0.0412	5.0613	-0.0417	0.0132	-0.1173	-0.1189
104	Prediction	4.883	4.9295	0.0465	-	-	0.0337	0.1338	0.1338
105	Training	5.233	5.0615	-0.1715	5.0592	-0.1738	0.0132	-0.4886	-0.4951
106	Prediction	5.025	4.9525	-0.0725	-	-	0.019	-0.207	-0.207
107	Training	4.445	5.0646	0.6196	5.0729	0.6279	0.0132	1.7651	1.7887
108	Training	4.71	5.0647	0.3547	5.0695	0.3595	0.0132	1.0106	1.0241
109	Prediction	4.546	4.9311	0.3851	-	-	0.0339	1.1087	1.1087
110	Training	4.46	4.956	0.496	4.9656	0.5056	0.019	1.4172	1.4447
111	Training	4.991	5.0606	0.0696	5.0615	0.0705	0.0132	0.1981	0.2008
112	Training	4.722	4.7052	-0.0168	4.7037	-0.0183	0.0828	-0.0496	-0.0541
113	Prediction	4.655	4.9554	0.3004	-	-	0.019	0.8581	0.8581
114	Training	5.05	4.9304	-0.1196	4.9262	-0.1238	0.0338	-0.3444	-0.3564
115	Training	4.835	5.0287	0.1937	5.0331	0.1981	0.0226	0.5543	0.5672
116	Training	5.105	5.137	0.032	5.1375	0.0325	0.0157	0.0914	0.0928
117	Prediction	4.872	5.0643	0.1923	-	-	0.0132	0.5479	0.5479
118	Training	4.767	4.9562	0.1892	4.9598	0.1928	0.019	0.5405	0.5509
119	Training	5.024	5.2091	0.1851	5.2196	0.1956	0.0537	0.5385	0.5691
120	Training	4.989	5.0646	0.0756	5.0656	0.0766	0.0132	0.2152	0.2181
121	Training	5.107	5.5393	0.4323	5.5441	0.4371	0.0109	1.2302	1.2438
122	Training	5.306	5.5388	0.2328	5.5413	0.2353	0.0109	0.6623	0.6696
123	Training	5.917	5.5385	-0.3785	5.5343	-0.3827	0.0109	-1.077	-1.0889
124	Training	5.578	5.5389	-0.0391	5.5385	-0.0395	0.0109	-0.1111	-0.1124
125	Training	5.078	5.5384	0.4604	5.5435	0.4655	0.0109	1.31	1.3244
126	Training	5.674	5.6219	-0.0521	5.6209	-0.0531	0.0174	-0.1488	-0.1515
127	Training	6.959	6.0442	-0.9148	6.011	-0.948	0.035	-2.6353	-2.731
128	Training	5.979	5.9936	0.0146	5.9941	0.0151	0.0341	0.042	0.0435
129	Training	6.357	5.8895	-0.4675	5.878	-0.479	0.0239	-1.3392	-1.372
130	Training	6.387	5.8895	-0.4975	5.8773	-0.5097	0.0239	-1.4251	-1.4601
131	Training	5.558	5.3619	-0.1961	5.3596	-0.1984	0.0114	-0.5581	-0.5646
132	Training	5.793	5.9342	0.1412	5.9379	0.1449	0.0256	0.4047	0.4154
133	Training	5.489	5.9352	0.4462	5.9469	0.4579	0.0256	1.2792	1.3129
134	Training	5.65	5.9137	0.2637	5.9217	0.2717	0.0296	0.7575	0.7806
135	Training	5.358	5.9142	0.5562	5.9312	0.5732	0.0296	1.598	1.6468
136	Training	5.561	5.9345	0.3735	5.9443	0.3833	0.0256	1.0708	1.099
137	Training	6.721	6.04	-0.681	6.0154	-0.7056	0.0349	-1.9617	-2.0326
138	Training	6.62	6.3608	-0.2592	6.3377	-0.2823	0.0819	-0.7655	-0.8337
139	Training	5.936	5.9195	-0.0165	5.919	-0.017	0.0305	-0.0475	-0.049
140	Prediction	5.59	5.9321	0.3421	-	-	0.0255	0.9808	0.9808
141	Training	5.272	5.6101	0.3381	5.6157	0.3437	0.0161	0.9646	0.9804

142	Training	6.456	6.0391	-0.4169	6.024	-0.432	0.0348	-1.201	-1.2443
143	Prediction	6.252	6.0402	-0.2118	-	-	0.0349	-0.6102	-0.6102
144	Training	6.585	6.0402	-0.5448	6.0205	-0.5645	0.0349	-1.5695	-1.6262
145	Training	5.815	5.7196	-0.0954	5.7181	-0.0969	0.0163	-0.2721	-0.2766
146	Prediction	5.539	5.7181	0.1791	-	-	0.0162	0.5109	0.5109
147	Training	5.064	5.7181	0.6541	5.7288	0.6648	0.0162	1.8661	1.8967
148	Training	5.212	5.0597	-0.1523	5.0577	-0.1543	0.0132	-0.4339	-0.4397
149	Prediction	5.523	4.931	-0.592	-	-	0.0338	-1.7043	-1.7043
150	Training	5.319	5.0179	-0.3011	5.003	-0.316	0.0472	-0.8729	-0.9161
151	Training	5.701	5.2197	-0.4813	5.2067	-0.4943	0.0264	-1.3803	-1.4178
152	Training	5.243	5.0223	-0.2207	5.015	-0.228	0.0319	-0.6348	-0.6557
153	Prediction	5.544	5.1087	-0.4353	-	-	0.0374	-1.2557	-1.2557
154	Prediction	7.097	5.9768	-1.1202	-	-	0.0535	-3.2585	-3.2585
155	Training	6.108	5.4577	-0.6503	5.4505	-0.6575	0.011	-1.8503	-1.8708
156	Training	4.066	5.2386	1.1726	5.2568	1.1908	0.0153	3.3439	3.3958
157	Training	5.229	5.8614	0.6324	5.8888	0.6598	0.0415	1.828	1.907
158	Training	6.367	5.704	-0.663	5.6539	-0.7131	0.0704	-1.9458	-2.093
159	Training	5.157	5.6955	0.5385	5.7104	0.5534	0.027	1.5448	1.5877
160	Prediction	4.658	5.6044	0.9464	-	-	0.0265	2.7144	2.7144
161	Training	5.147	5.3314	0.1844	5.3396	0.1926	0.0428	0.5332	0.557
162	Training	5.305	5.1766	-0.1284	5.1735	-0.1315	0.0233	-0.3678	-0.3765
163	Training	5.092	5.1754	0.0834	5.1773	0.0853	0.0233	0.2387	0.2444
164	Training	5.783	5.1783	-0.6047	5.1638	-0.6192	0.0234	-1.7315	-1.7731
165	Prediction	5.377	5.1771	-0.1999	-	-	0.0234	-0.5724	-0.5724
166	Training	5.09	5.1755	0.0855	5.1776	0.0876	0.0233	0.245	0.2508
167	Prediction	5.498	5.1761	-0.3219	-	-	0.0233	-0.9218	-0.9218
168	Training	5.313	5.1777	-0.1353	5.1745	-0.1385	0.0234	-0.3873	-0.3966
169	Prediction	5.035	5.1773	0.1423	-	-	0.0234	0.4075	0.4075
170	Training	7.301	7.1776	-0.1234	7.1696	-0.1314	0.0611	-0.3604	-0.3838
171	Training	7	7.1782	0.1782	7.1898	0.1898	0.0611	0.5203	0.5542
172	Training	7.398	7.179	-0.219	7.1648	-0.2332	0.0611	-0.6394	-0.6811
173	Training	6.921	7.1787	0.2577	7.1954	0.2744	0.0611	0.7525	0.8015
174	Training	7.155	7.1777	0.0227	7.1792	0.0242	0.0611	0.0663	0.0706
175	Prediction	7.155	7.178	0.023	-	-	0.0611	0.0673	0.0673
176	Training	7	7.1772	0.1772	7.1887	0.1887	0.0611	0.5175	0.5512
177	Training	7.155	7.1779	0.0229	7.1794	0.0244	0.0611	0.0668	0.0711
178	Training	7.155	7.1767	0.0217	7.1781	0.0231	0.0612	0.0634	0.0676
179	Training	7.222	7.2629	0.0409	7.2656	0.0436	0.0622	0.1196	0.1275
180	Training	7.301	7.263	-0.038	7.2604	-0.0406	0.0622	-0.1111	-0.1185
181	Training	7.301	7.177	-0.124	7.169	-0.132	0.0611	-0.3621	-0.3857
182	Training	7.222	7.1784	-0.0436	7.1755	-0.0465	0.0611	-0.1275	-0.1358
183	Training	7.301	6.2533	-1.0477	6.1675	-1.1335	0.0758	-3.0838	-3.3366
184	Training	7	7.341	0.341	7.3665	0.3665	0.0694	1.0004	1.0751
185	Training	7.301	7.1774	-0.1236	7.1694	-0.1316	0.0611	-0.3609	-0.3844
186	Prediction	7.097	7.1787	0.0817	-	-	0.0611	0.2385	0.2385
187	Prediction	7.155	6.8214	-0.3336	-	-	0.1084	-0.9998	-0.9998
188	Prediction	7.097	6.8204	-0.2766	-	-	0.1084	-0.829	-0.829
189	Training	6.959	6.799	-0.16	6.7744	-0.1846	0.1335	-0.4863	-0.5612
190	Training	6.585	6.8013	0.2163	6.8346	0.2496	0.1335	0.6576	0.7589
191	Training	6.638	6.7988	0.1608	6.8236	0.1856	0.1335	0.489	0.5643
192	Training	6.886	6.7991	-0.0869	6.7857	-0.1003	0.1335	-0.2641	-0.3048

193	Training	6.796	6.7989	0.0029	6.7993	0.0033	0.1335	0.0088	0.0102
194	Prediction	4.648	5.1699	0.5219	-	-	0.0183	1.4906	1.4906
195	Training	4.831	4.991	0.16	4.9936	0.1626	0.0159	0.4563	0.4637
196	Training	4.776	5.1697	0.3937	5.177	0.401	0.0183	1.1243	1.1453
197	Training	4.429	4.9549	0.5259	4.9726	0.5436	0.0326	1.5131	1.5641
198	Training	5.12	5.1685	0.0485	5.1694	0.0494	0.0184	0.1385	0.1411
199	Training	5.229	4.9908	-0.2382	4.9869	-0.2421	0.0159	-0.6796	-0.6906
200	Prediction	5.026	5.1683	0.1423	-	-	0.0184	0.4064	0.4064
201	Training	5.042	5.1705	0.1285	5.1729	0.1309	0.0182	0.367	0.3738
202	Training	5.303	4.9915	-0.3115	4.9865	-0.3165	0.0159	-0.8885	-0.9028
203	Training	4.645	5.1686	0.5236	5.1784	0.5334	0.0184	1.4955	1.5234
204	Training	5.051	4.9914	-0.0596	4.9904	-0.0606	0.0159	-0.1701	-0.1728
205	Training	5.554	5.1709	-0.3831	5.1638	-0.3902	0.0182	-1.094	-1.1143
206	Training	4.957	4.9912	0.0342	4.9918	0.0348	0.0159	0.0976	0.0992
207	Training	5.194	5.1696	-0.0244	5.1691	-0.0249	0.0183	-0.0697	-0.071
208	Training	4.844	4.9903	0.1463	4.9927	0.1487	0.016	0.4175	0.4242
209	Training	4.575	5.2532	0.6782	5.2637	0.6887	0.0152	1.934	1.9639
210	Training	5.324	5.0753	-0.2487	5.072	-0.252	0.0133	-0.7084	-0.718
211	Training	5.228	4.9912	-0.2368	4.9873	-0.2407	0.0159	-0.6756	-0.6865
212	Training	5.301	5.1691	-0.1319	5.1666	-0.1344	0.0183	-0.3767	-0.3838
213	Training	5.682	4.9912	-0.6908	4.9801	-0.7019	0.0159	-1.9705	-2.0023
214	Training	5.745	5.1696	-0.5754	5.1589	-0.5861	0.0183	-1.6433	-1.6739
215	Prediction	5.412	5.1696	-0.2424	-	-	0.0183	-0.6923	-0.6923
216	Training	5.353	4.9918	-0.3612	4.9859	-0.3671	0.0159	-1.0305	-1.0471
217	Training	5.205	5.2562	0.0512	5.257	0.052	0.0152	0.1459	0.1482
218	Training	4.485	5.0748	0.5898	5.0827	0.5977	0.0133	1.6802	1.7027
219	Training	5.143	4.6572	-0.4858	4.6074	-0.5356	0.093	-1.4434	-1.5914

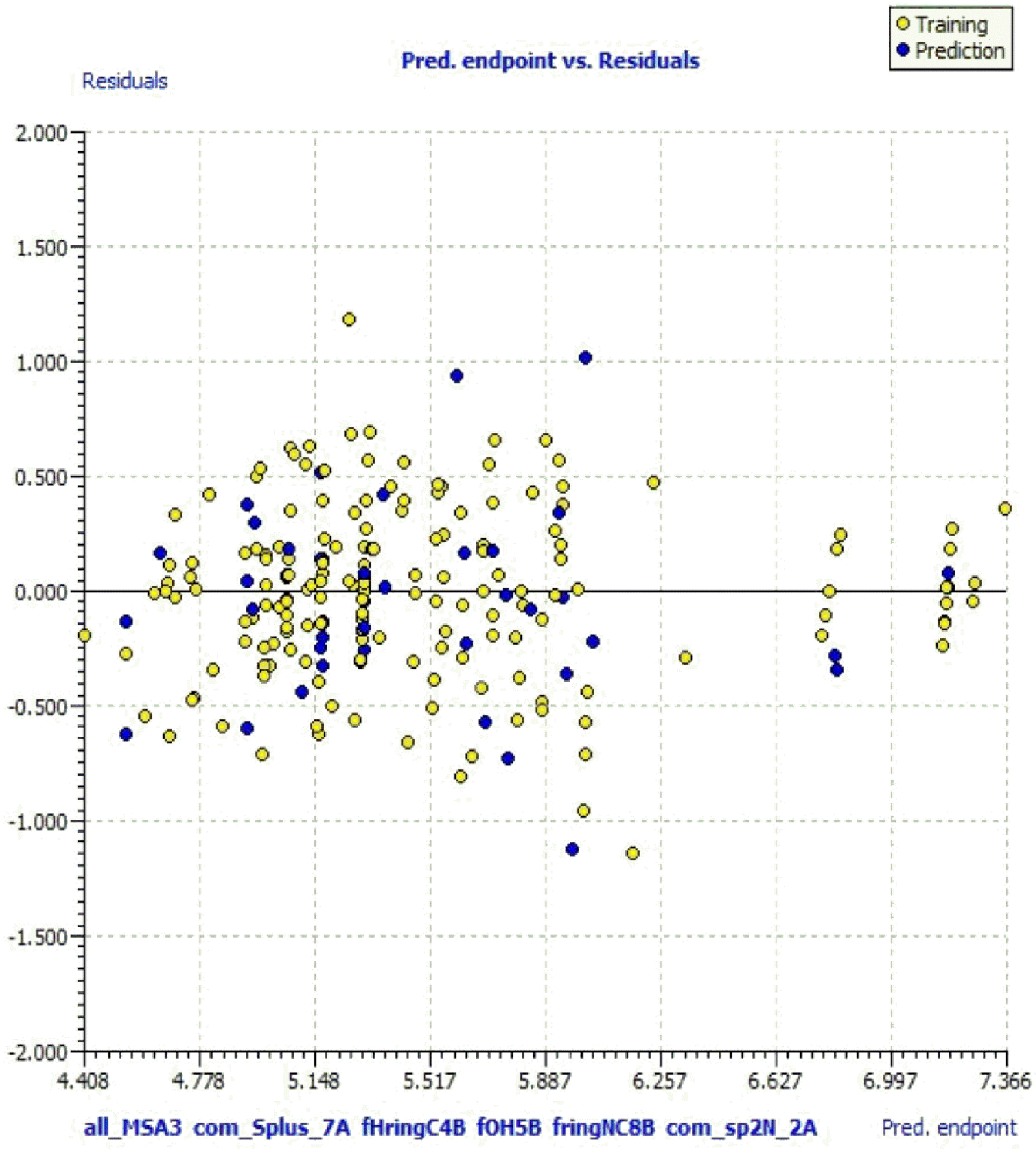
Training
Prediction
Pred. endpoint vs. Residuals
Residuals
2.000
1.500
1.000
0.500
0.000
-0.500
-1.000
-1.500
-2.000
4.408
4.778
5.148
5.517
5.887
6.257
6.627
6.997
7.366
all_MSA3 com_Splus_7A fHringC4B fOH5B fringNC8B com_sp2N_2A
Pred. endpoint

(a)

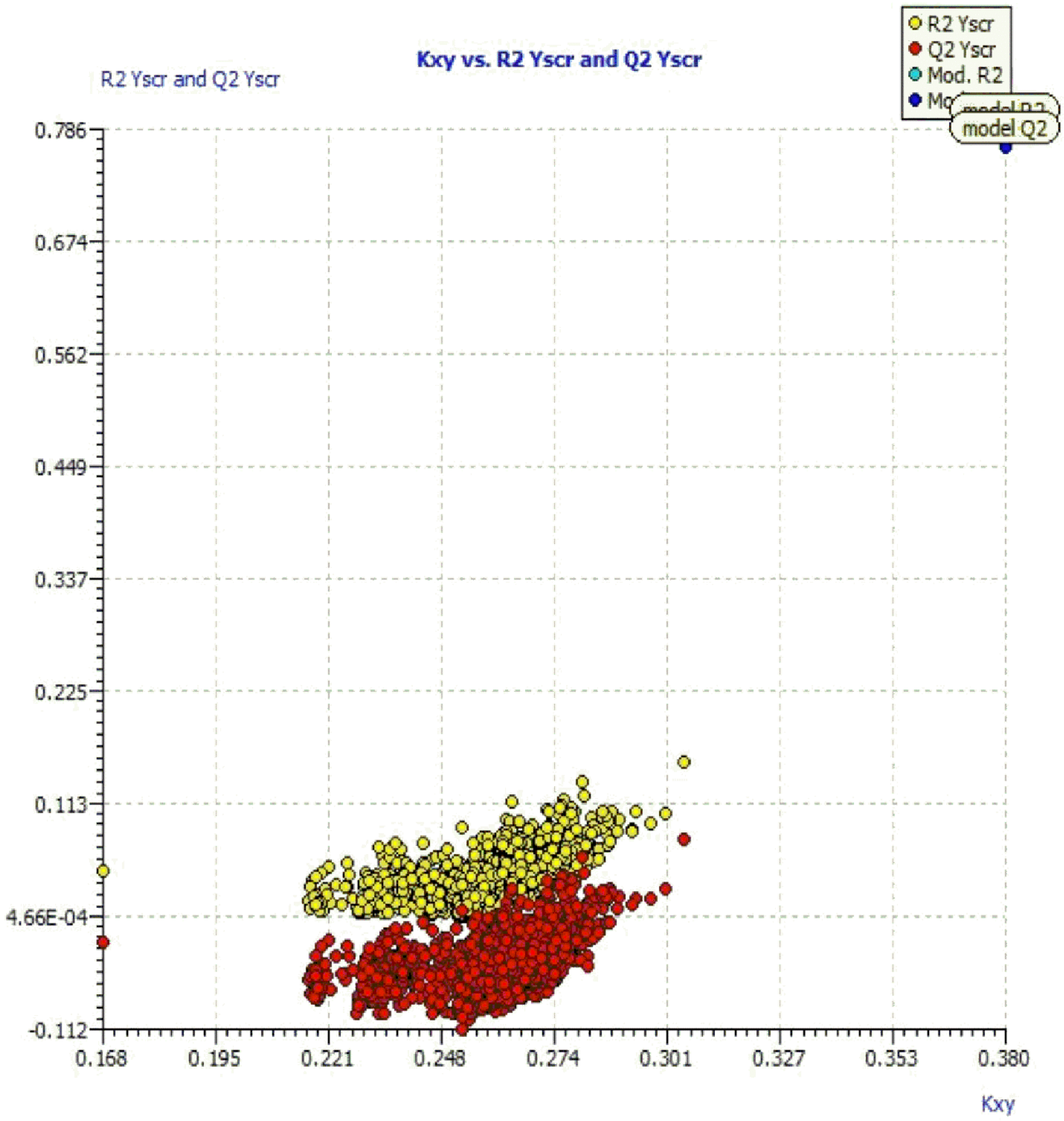

(b)

Figure S1: Different graphs associated with model 1.1 (a) graph of pred. endpoint vs. residual values (b) Y-scrambling plot

Table S2: Details regarding performance of model 1.2

ID	Status	Exp. endpoint	Pred. by model eq.	Pred. Mod.Eq.Res.	Pred. LOO	Pred. LOO Res.	HAT i/i (h*=0.1186)	Std.Pred .Mod.Eq . Res.	Std.Pred .LOO Res.
1	Training	5.664	5.7992	0.1352	5.8021	0.1381	0.0206	0.3866	0.3947
2	Training	6	5.9406	-0.0594	5.9377	-0.0623	0.0453	-0.1721	-0.1803
3	Training	6.174	5.8736	-0.3004	5.864	-0.31	0.0311	-0.8634	-0.8911

4	Prediction	6.31	6.0029	-0.3071	-	-	0.0287	-0.8816	-0.8816
5	Prediction	5.971	5.9953	0.0243	-	-	0.0282	0.0697	0.0697
6	Training	6.357	5.8752	-0.4818	5.8596	-0.4974	0.0314	-1.3849	-1.4297
7	Prediction	6.495	5.932	-0.563	-	-	0.0535	-1.6373	-1.6373
8	Training	5.093	5.626	0.533	5.6383	0.5453	0.0225	1.5253	1.5603
9	Training	5.686	5.7673	0.0813	5.7712	0.0852	0.0455	0.2355	0.2468
10	Prediction	5.456	5.7061	0.2501	-	-	0.033	0.7195	0.7195
11	Training	5.728	5.979	0.251	5.986	0.258	0.0273	0.72	0.7402
12	Training	5.79	5.9805	0.1905	5.9858	0.1958	0.0274	0.5464	0.5618
13	Training	5.991	5.8589	-0.1321	5.8551	-0.1359	0.0286	-0.3791	-0.3902
14	Prediction	5.78	5.8317	0.0517	-	-	0.033	0.1487	0.1487
15	Training	5.073	5.5082	0.4352	5.5165	0.4435	0.0188	1.2429	1.2666
16	Training	5.738	5.6509	-0.0871	5.6486	-0.0894	0.0251	-0.2497	-0.2561
17	Training	5.879	5.87	-0.009	5.8698	-0.0092	0.0281	-0.0258	-0.0265
18	Training	5.807	5.86	0.053	5.8615	0.0545	0.0288	0.152	0.1565
19	Training	6.102	5.7489	-0.3531	5.7429	-0.3591	0.0167	-1.0073	-1.0244
20	Prediction	5.86	5.7071	-0.1529	-	-	0.0331	-0.4399	-0.4399
21	Training	5.115	5.2831	0.1681	5.2881	0.1731	0.0285	0.4826	0.4968
22	Training	4.928	5.3595	0.4315	5.3671	0.4391	0.0171	1.2315	1.2529
23	Training	5.189	5.2831	0.0941	5.2859	0.0969	0.0285	0.2702	0.2781
24	Training	5.471	5.2831	-0.1879	5.2776	-0.1934	0.0285	-0.5392	-0.5551
25	Prediction	5.556	5.2831	-0.2729	-	-	0.0285	-0.7832	-0.7832
26	Training	5.295	5.2831	-0.0119	5.2828	-0.0122	0.0285	-0.0341	-0.0351
27	Training	5.421	5.2831	-0.1379	5.2791	-0.1419	0.0285	-0.3957	-0.4073
28	Training	5.502	5.2831	-0.2189	5.2767	-0.2253	0.0285	-0.6282	-0.6467
29	Training	5.391	5.2831	-0.1079	5.28	-0.111	0.0285	-0.3096	-0.3187
30	Training	5.034	5.2831	0.2491	5.2905	0.2565	0.0285	0.7151	0.7361
31	Training	5.04	5.4057	0.3657	5.4105	0.3705	0.0131	1.0414	1.0552
32	Training	5.46	5.2831	-0.1769	5.2779	-0.1821	0.0285	-0.5077	-0.5226
33	Prediction	5.342	5.2831	-0.0589	-	-	0.0285	-0.169	-0.169
34	Prediction	5.354	5.3537	-0.0003	-	-	0.0178	-0.0009	-0.0009
35	Training	5.6	5.2831	-0.3169	5.2738	-0.3262	0.0285	-0.9095	-0.9362
36	Training	4.746	5.2831	0.5371	5.2989	0.5529	0.0285	1.5418	1.5871
37	Training	5.481	5.4477	-0.0333	5.4473	-0.0337	0.0113	-0.0947	-0.0958
38	Training	5.399	5.4479	0.0489	5.4485	0.0495	0.0113	0.1392	0.1408
39	Training	5.772	5.4447	-0.3273	5.4409	-0.3311	0.0114	-0.9313	-0.942
40	Prediction	5.243	5.2831	0.0401	-	-	0.0285	0.1152	0.1152
41	Training	5.91	5.6868	-0.2232	5.6703	-0.2397	0.0685	-0.6544	-0.7025
42	Training	4.914	5.2831	0.3691	5.294	0.38	0.0285	1.0596	1.0907
43	Training	5.298	5.2831	-0.0149	5.2827	-0.0153	0.0285	-0.0427	-0.0439
44	Training	5.264	5.2831	0.0191	5.2837	0.0197	0.0285	0.0549	0.0565
45	Prediction	5.456	5.2831	-0.1729	-	-	0.0285	-0.4962	-0.4962
46	Training	5.59	5.2831	-0.3069	5.2741	-0.3159	0.0285	-0.8808	-0.9067
47	Training	5.437	4.8795	-0.5575	4.8569	-0.5801	0.039	-1.6089	-1.6743
48	Training	5.339	5.3794	0.0404	5.3808	0.0418	0.0349	0.1163	0.1205
49	Prediction	5.22	5.3794	0.1594	-	-	0.0349	0.459	0.459

50	Prediction	4.942	5.4423	0.5003	-	-	0.024	1.4327	1.4327
51	Training	4.931	5.3519	0.4209	5.3616	0.4306	0.0225	1.2045	1.2322
52	Training	5.248	5.3696	0.1216	5.3773	0.1293	0.0597	0.3548	0.3773
53	Training	4.943	5.2621	0.3191	5.2894	0.3464	0.0789	0.9406	1.0212
54	Training	5.315	4.9429	-0.3721	4.8439	-0.4711	0.2102	-1.1845	-1.4998
55	Training	4.681	4.7835	0.1025	4.7922	0.1112	0.0784	0.3021	0.3278
56	Training	4.556	5.0893	0.5333	5.1407	0.5847	0.0879	1.5799	1.7322
57	Prediction	4.674	4.5118	-0.1622	-	-	0.0801	-0.4784	-0.4784
58	Training	4.633	4.6293	-0.0037	4.629	-0.004	0.0774	-0.0108	-0.0117
59	Training	4.361	4.6304	0.2694	4.6529	0.2919	0.0773	0.7934	0.8598
60	Prediction	4.482	4.7083	0.2263	-	-	0.0779	0.6669	0.6669
61	Training	4.756	4.8033	0.0473	4.8077	0.0517	0.0864	0.1399	0.1532
62	Training	4.623	4.7848	0.1618	4.7985	0.1755	0.0784	0.4767	0.5172
63	Training	4.814	4.6251	-0.1889	4.6092	-0.2048	0.0775	-0.5565	-0.6032
64	Prediction	5.163	4.6066	-0.5564	-	-	0.0814	-1.6424	-1.6424
65	Training	4.566	4.7259	0.1599	4.7395	0.1735	0.0785	0.4711	0.5113
66	Training	4.673	4.63	-0.043	4.6264	-0.0466	0.0773	-0.1266	-0.1372
67	Training	4.762	4.8628	0.1008	4.8721	0.1101	0.0842	0.298	0.3254
68	Training	5.223	4.7651	-0.4579	4.7148	-0.5082	0.0991	-1.3647	-1.5148
69	Training	4.634	5.2071	0.5731	5.2384	0.6044	0.0518	1.665	1.756
70	Training	5.143	5.3288	0.1858	5.339	0.196	0.052	0.5398	0.5694
71	Training	4.87	5.3069	0.4369	5.3282	0.4582	0.0463	1.2658	1.3273
72	Training	6.027	5.5023	-0.5247	5.4961	-0.5309	0.0117	-1.4931	-1.5108
73	Training	6.42	5.6015	-0.8185	5.5847	-0.8353	0.0202	-2.3394	-2.3875
74	Training	5.91	5.6026	-0.3074	5.5962	-0.3138	0.0203	-0.8787	-0.8969
75	Training	5.415	5.7967	0.3817	5.8056	0.3906	0.0228	1.0923	1.1178
76	Prediction	5.914	5.8979	-0.0161	-	-	0.0228	-0.0461	-0.0461
77	Training	5.314	5.5312	0.2172	5.5341	0.2201	0.0132	0.6185	0.6268
78	Training	5.754	6.1368	0.3828	6.1647	0.4107	0.0677	1.1218	1.2033
79	Training	5.793	5.5302	-0.2628	5.5268	-0.2662	0.0131	-0.7483	-0.7582
80	Prediction	4.991	5.961	0.97	-	-	0.0373	2.797	2.797
81	Prediction	6.26	5.7605	-0.4995	-	-	0.0307	-1.4353	-1.4353
82	Training	5.489	5.3777	-0.1113	5.3754	-0.1136	0.0201	-0.3182	-0.3247
83	Training	5.333	5.2833	-0.0497	5.2825	-0.0505	0.0148	-0.1417	-0.1439
84	Training	5.116	5.2251	0.1091	5.2273	0.1113	0.0202	0.3118	0.3183
85	Training	5.511	5.3772	-0.1338	5.3744	-0.1366	0.02	-0.3825	-0.3903
86	Training	5.83	5.2823	-0.5477	5.2742	-0.5558	0.0147	-1.5609	-1.5843
87	Training	5.499	5.5489	0.0499	5.551	0.052	0.0404	0.1441	0.1502
88	Training	5.274	5.1299	-0.1441	5.1281	-0.1459	0.0122	-0.4103	-0.4153
89	Training	5.423	5.1278	-0.2952	5.1241	-0.2989	0.0123	-0.8403	-0.8508
90	Training	4.594	4.1966	-0.3974	4.1273	-0.4667	0.1486	-1.2184	-1.431
91	Training	4.388	4.9007	0.5127	4.9669	0.5789	0.1144	1.5413	1.7404
92	Training	5.164	4.9832	-0.1808	4.9627	-0.2013	0.1015	-0.5398	-0.6007
93	Training	4.641	4.6848	0.0438	4.6887	0.0477	0.0814	0.1294	0.1408
94	Training	4.494	5.0261	0.5321	5.0512	0.5572	0.0451	1.5404	1.6131
95	Training	4.916	4.9729	0.0569	4.9743	0.0583	0.0239	0.1629	0.1669

96	Training	5.1	4.9546	-0.1454	4.9494	-0.1506	0.0346	-0.4187	-0.4337
97	Training	5.159	4.9708	-0.1882	4.9662	-0.1928	0.0238	-0.5388	-0.552
98	Training	5.063	4.9663	-0.0967	4.9644	-0.0986	0.0187	-0.2763	-0.2816
99	Training	5.226	4.7125	-0.5135	4.6869	-0.5391	0.0476	-1.4885	-1.5628
100	Prediction	4.994	4.9717	-0.0223	-	-	0.0238	-0.0638	-0.0638
101	Training	5.139	4.8536	-0.2854	4.8404	-0.2986	0.044	-0.8259	-0.864
102	Training	5.09	4.9717	-0.1183	4.9688	-0.1212	0.0238	-0.3388	-0.3471
103	Training	5.103	4.9722	-0.1308	4.969	-0.134	0.0239	-0.3746	-0.3837
104	Prediction	4.883	4.8512	-0.0318	-	-	0.0437	-0.0921	-0.0921
105	Training	5.233	4.9719	-0.2611	4.9655	-0.2675	0.0238	-0.7477	-0.766
106	Prediction	5.025	4.9657	-0.0593	-	-	0.0187	-0.1693	-0.1693
107	Training	4.445	4.8787	0.4337	4.9032	0.4582	0.0535	1.2612	1.3325
108	Training	4.71	4.9751	0.2651	4.9815	0.2715	0.0239	0.759	0.7776
109	Prediction	4.546	4.8527	0.3067	-	-	0.0439	0.8875	0.8875
110	Training	4.46	4.9691	0.5091	4.9789	0.5189	0.0188	1.4541	1.4819
111	Training	4.991	4.971	-0.02	4.9705	-0.0205	0.0238	-0.0574	-0.0588
112	Training	4.722	4.6533	-0.0687	4.6466	-0.0754	0.0882	-0.2036	-0.2233
113	Prediction	4.655	4.8722	0.2172	-	-	0.0287	0.6236	0.6236
114	Training	5.05	4.852	-0.198	4.843	-0.207	0.0438	-0.5727	-0.599
115	Training	4.835	4.944	0.109	4.9478	0.1128	0.0335	0.3136	0.3245
116	Training	5.105	5.0458	-0.0592	5.0441	-0.0609	0.0275	-0.1698	-0.1746
117	Prediction	4.872	4.8784	0.0064	-	-	0.0535	0.0187	0.0187
118	Training	4.767	4.873	0.106	4.8762	0.1092	0.0288	0.3044	0.3134
119	Training	5.024	5.1206	0.0966	5.1276	0.1036	0.0674	0.283	0.3034
120	Training	4.989	4.8786	-0.1104	4.8724	-0.1166	0.0535	-0.321	-0.3391
121	Training	5.107	5.6085	0.5015	5.616	0.509	0.0147	1.4294	1.4507
122	Training	5.306	5.6079	0.3019	5.6124	0.3064	0.0147	0.8606	0.8734
123	Training	5.917	5.6077	-0.3093	5.6031	-0.3139	0.0147	-0.8816	-0.8947
124	Training	5.578	5.5119	-0.0661	5.5111	-0.0669	0.0121	-0.1882	-0.1905
125	Training	5.078	5.5113	0.4333	5.5166	0.4386	0.0121	1.2334	1.2485
126	Training	5.674	5.6893	0.0153	5.6896	0.0156	0.0203	0.0437	0.0446
127	Training	6.959	6.0857	-0.8733	6.0525	-0.9065	0.0366	-2.5172	-2.6127
128	Training	5.979	5.9399	-0.0391	5.9384	-0.0406	0.0369	-0.1127	-0.117
129	Training	6.357	5.8422	-0.5148	5.8282	-0.5288	0.0264	-1.476	-1.5161
130	Training	6.387	5.8422	-0.5448	5.8274	-0.5596	0.0264	-1.562	-1.6044
131	Training	5.558	5.4499	-0.1081	5.4481	-0.1099	0.0168	-0.3084	-0.3137
132	Training	5.793	5.886	0.093	5.8887	0.0957	0.0284	0.2669	0.2747
133	Training	5.489	5.887	0.398	5.8987	0.4097	0.0285	1.1424	1.1759
134	Training	5.65	5.8702	0.2202	5.8776	0.2276	0.0326	0.6333	0.6547
135	Training	5.358	5.8707	0.5127	5.8881	0.5301	0.0327	1.4749	1.5248
136	Training	5.561	5.8863	0.3253	5.8959	0.3349	0.0284	0.9338	0.9611
137	Training	6.721	5.9853	-0.7357	5.9561	-0.7649	0.0382	-2.1223	-2.2065
138	Training	6.62	6.2866	-0.3334	6.2549	-0.3651	0.0867	-0.9871	-1.0807
139	Training	5.936	5.8758	-0.0602	5.8737	-0.0623	0.0336	-0.1731	-0.1792
140	Prediction	5.59	5.884	0.294	-	-	0.0283	0.8438	0.8438
141	Training	5.272	5.5815	0.3095	5.5871	0.3151	0.0177	0.8836	0.8995

142	Training	6.456	5.9844	-0.4716	5.9657	-0.4903	0.0381	-1.3604	-1.4143
143	Prediction	6.252	6.0817	-0.1703	-	-	0.0364	-0.4908	-0.4908
144	Training	6.585	6.0817	-0.5033	6.0627	-0.5223	0.0364	-1.4506	-1.5054
145	Training	5.815	5.6845	-0.1305	5.6821	-0.1329	0.0183	-0.3726	-0.3796
146	Prediction	5.539	5.683	0.144	-	-	0.0181	0.411	0.411
147	Training	5.064	5.683	0.619	5.6944	0.6304	0.0181	1.7672	1.7998
148	Training	5.212	5.2589	0.0469	5.2612	0.0492	0.0482	0.1359	0.1428
149	Prediction	5.523	5.1414	-0.3816	-	-	0.0687	-1.1187	-1.1187
150	Training	5.319	5.2265	-0.0925	5.2185	-0.1005	0.0791	-0.2728	-0.2963
151	Training	5.701	5.4047	-0.2963	5.3873	-0.3137	0.0553	-0.8626	-0.9131
152	Training	5.243	5.2157	-0.0273	5.2138	-0.0292	0.0649	-0.08	-0.0856
153	Prediction	5.544	5.3002	-0.2438	-	-	0.0677	-0.7143	-0.7143
154	Prediction	7.097	6.0305	-1.0665	-	-	0.0558	-3.1052	-3.1052
155	Training	6.108	5.6249	-0.4831	5.6056	-0.5024	0.0384	-1.3938	-1.4494
156	Training	4.066	5.2264	1.1604	5.2448	1.1788	0.0156	3.3089	3.3614
157	Training	5.229	5.9261	0.6971	5.9578	0.7288	0.0435	2.0165	2.1081
158	Training	6.367	5.7829	-0.5841	5.736	-0.631	0.0743	-1.7176	-1.8554
159	Training	5.157	5.8642	0.7072	5.9017	0.7447	0.0504	2.0531	2.1622
160	Prediction	4.658	5.6637	1.0057	-	-	0.0309	2.8902	2.8902
161	Training	5.147	5.5139	0.3669	5.5408	0.3938	0.0683	1.0754	1.1543
162	Training	5.305	5.3624	0.0574	5.3657	0.0607	0.0535	0.167	0.1765
163	Training	5.092	5.3612	0.2692	5.3765	0.2845	0.0535	0.783	0.8272
164	Training	5.783	5.3642	-0.4188	5.3404	-0.4426	0.0536	-1.218	-1.287
165	Prediction	5.377	5.3629	-0.0141	-	-	0.0535	-0.0409	-0.0409
166	Training	5.09	5.3614	0.2714	5.3768	0.2868	0.0535	0.7893	0.8339
167	Prediction	5.498	5.3619	-0.1361	-	-	0.0535	-0.3957	-0.3957
168	Training	5.313	5.3636	0.0506	5.3664	0.0534	0.0536	0.1471	0.1554
169	Prediction	5.035	5.3631	0.3281	-	-	0.0536	0.9542	0.9542
170	Training	7.301	7.0819	-0.2191	7.0699	-0.2311	0.0521	-0.6367	-0.6717
171	Training	7	7.0824	0.0824	7.087	0.087	0.0521	0.2396	0.2527
172	Training	7.398	7.0833	-0.3147	7.066	-0.332	0.0521	-0.9144	-0.9647
173	Training	6.921	7.0829	0.1619	7.0918	0.1708	0.0521	0.4705	0.4964
174	Training	7.155	7.082	-0.073	7.078	-0.077	0.0521	-0.2122	-0.2238
175	Prediction	7.155	7.0823	-0.0727	-	-	0.0521	-0.2112	-0.2112
176	Training	7	7.0815	0.0815	7.086	0.086	0.0521	0.2368	0.2498
177	Training	7.155	7.0822	-0.0728	7.0781	-0.0769	0.0521	-0.2117	-0.2233
178	Training	7.155	7.081	-0.074	7.077	-0.078	0.0521	-0.2149	-0.2268
179	Training	7.222	7.1654	-0.0566	7.1622	-0.0598	0.0543	-0.1646	-0.1741
180	Training	7.301	7.1654	-0.1356	7.1577	-0.1433	0.0543	-0.3943	-0.417
181	Training	7.301	7.0813	-0.2197	7.0693	-0.2317	0.0521	-0.6383	-0.6734
182	Training	7.222	7.0826	-0.1394	7.075	-0.147	0.0521	-0.405	-0.4272
183	Training	7.301	6.1134	-1.1876	6.0767	-1.2243	0.03	-3.4115	-3.5169
184	Training	7	7.2418	0.2418	7.2579	0.2579	0.0624	0.7066	0.7537
185	Training	7.301	7.0817	-0.2193	7.0697	-0.2313	0.0521	-0.6372	-0.6722
186	Prediction	7.097	7.0829	-0.0141	-	-	0.0521	-0.0408	-0.0408
187	Prediction	7.155	6.7634	-0.3916	-	-	0.102	-1.1692	-1.1692

188	Prediction	7.097	6.7624	-0.3346	-	-	0.102	-0.999	-0.999
189	Training	6.959	7.0824	0.1234	7.0892	0.1302	0.0521	0.3587	0.3784
190	Training	6.585	7.0847	0.4997	7.1121	0.5271	0.0521	1.4519	1.5316
191	Training	6.638	7.0823	0.4443	7.1067	0.4687	0.0521	1.2909	1.3619
192	Training	6.886	7.0825	0.1965	7.0933	0.2073	0.0521	0.5711	0.6024
193	Training	6.796	7.0823	0.2863	7.098	0.302	0.0521	0.832	0.8777
194	Prediction	4.648	5.1592	0.5112	-	-	0.0184	1.4597	1.4597
195	Training	4.831	4.9991	0.1681	5.0018	0.1708	0.0158	0.4794	0.4871
196	Training	4.776	5.1589	0.3829	5.1661	0.3901	0.0185	1.0935	1.1141
197	Training	4.429	4.9572	0.5282	4.975	0.546	0.0326	1.5194	1.5705
198	Training	5.12	5.1578	0.0378	5.1585	0.0385	0.0186	0.1079	0.11
199	Training	5.229	4.9989	-0.2301	4.9952	-0.2338	0.0158	-0.6562	-0.6668
200	Prediction	5.026	5.1576	0.1316	-	-	0.0186	0.3758	0.3758
201	Training	5.042	5.1598	0.1178	5.162	0.12	0.0184	0.3363	0.3426
202	Training	5.303	4.9997	-0.3033	4.9948	-0.3082	0.0158	-0.865	-0.8789
203	Training	4.645	5.1579	0.5129	5.1676	0.5226	0.0185	1.4647	1.4923
204	Training	5.051	4.9995	-0.0515	4.9987	-0.0523	0.0158	-0.1469	-0.1492
205	Training	5.554	5.1602	-0.3938	5.1528	-0.4012	0.0184	-1.1246	-1.1456
206	Training	4.957	4.9993	0.0423	5	0.043	0.0158	0.1207	0.1227
207	Training	5.194	5.1589	-0.0351	5.1582	-0.0358	0.0185	-0.1003	-0.1022
208	Training	4.844	4.9985	0.1545	5.001	0.157	0.0159	0.4406	0.4477
209	Training	4.575	5.2407	0.6657	5.2512	0.6762	0.0156	1.8982	1.9283
210	Training	5.324	5.0817	-0.2423	5.0784	-0.2456	0.0131	-0.6902	-0.6993
211	Training	5.228	4.9993	-0.2287	4.9956	-0.2324	0.0158	-0.6523	-0.6627
212	Training	5.301	5.1584	-0.1426	5.1557	-0.1453	0.0185	-0.4073	-0.415
213	Training	5.682	4.9994	-0.6826	4.9884	-0.6936	0.0158	-1.9467	-1.978
214	Training	5.745	5.1589	-0.5861	5.1479	-0.5971	0.0185	-1.6738	-1.7053
215	Prediction	5.412	5.1589	-0.2531	-	-	0.0185	-0.7229	-0.7229
216	Training	5.353	4.9999	-0.3531	4.9942	-0.3588	0.0158	-1.007	-1.0232
217	Training	5.205	5.2436	0.0386	5.2442	0.0392	0.0156	0.1101	0.1118
218	Training	4.485	5.0811	0.5961	5.089	0.604	0.0131	1.6977	1.7203
219	Training	5.143	4.8151	-0.3279	4.7732	-0.3698	0.1132	-0.9852	-1.111

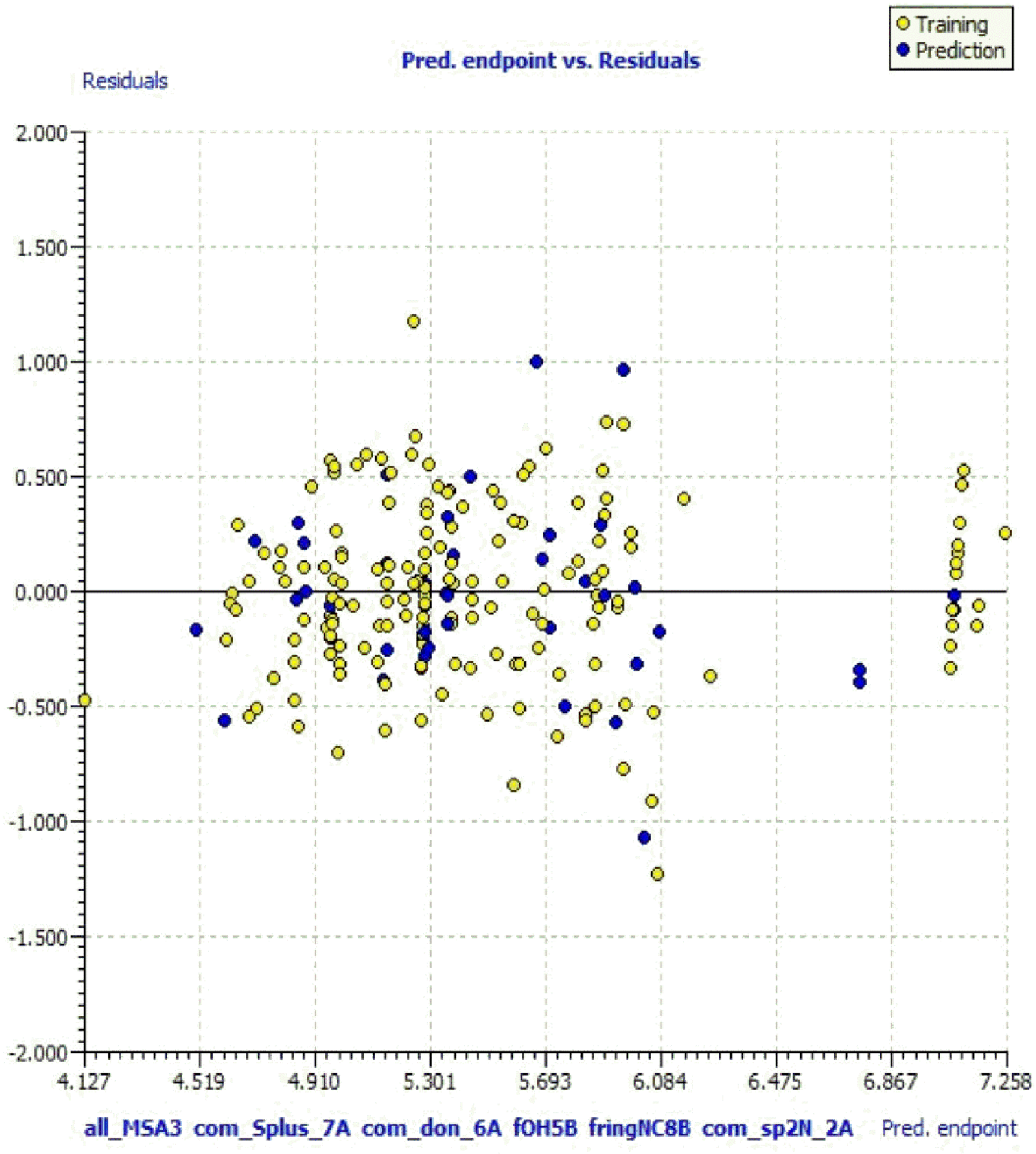
Training
Prediction
Pred. endpoint vs. Residuals
Residuals
2.000
1.500
1.000
0.500
0.000
-0.500
-1.000
-1.500
-2.000
4.127
4.519
4.910
5.301
5.693
6.084
6.475
6.867
7.258
all_MSA3 com_Splus_7A com_don_6A fOH5B fringNC8B com_sp2N_2A
Pred. endpoint

(a)

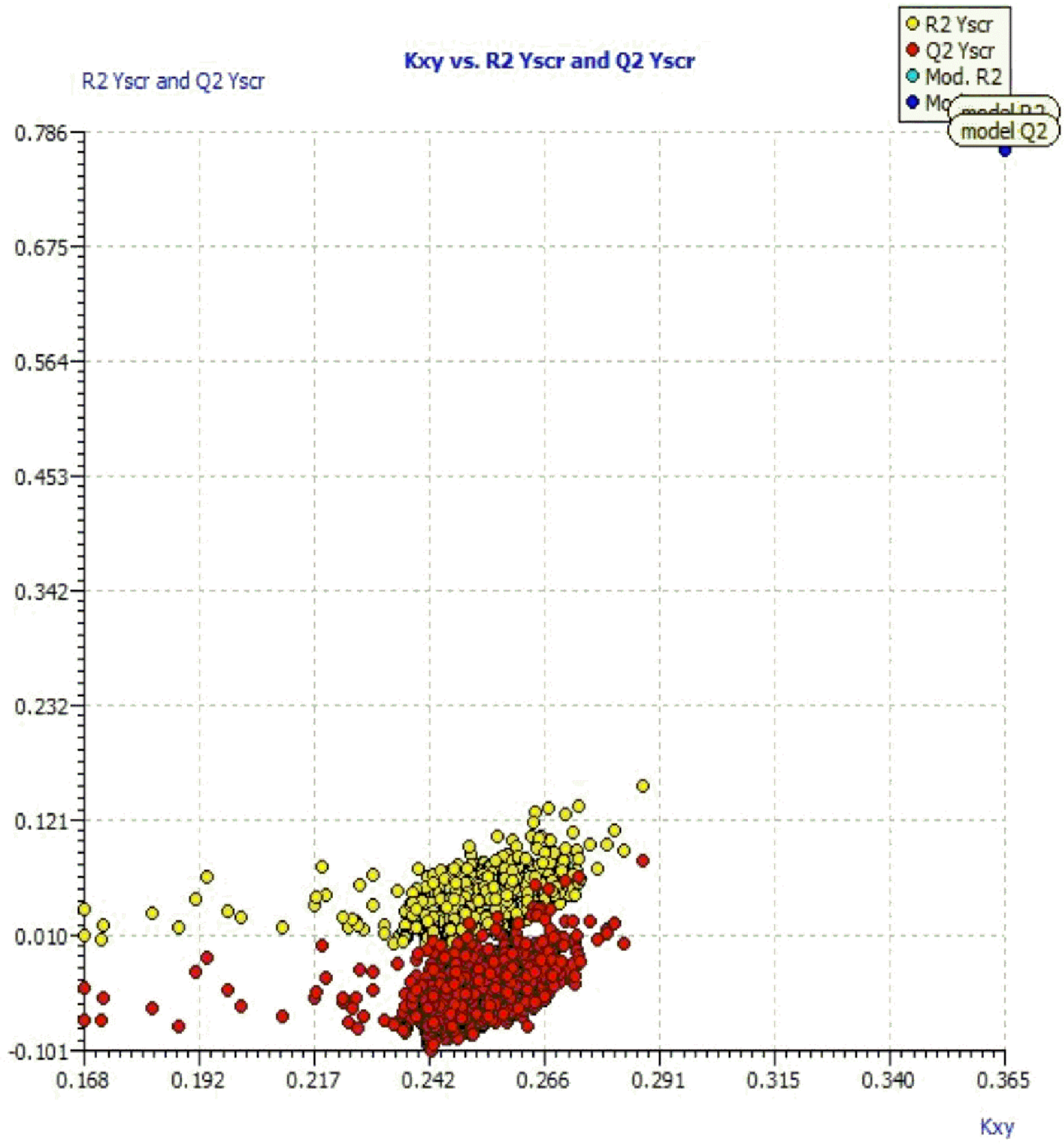

(b)

Figure S2: Different graphs associated with model 1.2 (a) graph of pred. endpoint vs. residual values (b) Y-scrambling plot:

Table S3: The values for selected molecular descriptors present in QSAR models.

No.	pIC_{50}	all_MSA3	com_Splus_7A	fHringC4B	fOH5B	fringNC8B	com_sp2N_2A	com_don_6A
1	5.664	25.731	0	0	5	0	0	1
2	6	37.134	0	0	5	0	0	1
3	6.174	31.731	0	0	5	0	0	1
4	6.31	25.880	0	0	7	0	0	1
5	5.971	25.268	0	0	7	0	0	1
6	6.357	31.863	0	0	5	0	0	1

7	6.495	25.267	0	0	7	1	0	0
8	5.093	24.631	0	0	5	1	0	1
9	5.686	36.031	0	0	5	1	0	1
10	5.456	31.090	0	0	5	1	0	1
11	5.728	23.952	0	0	7	0	0	1
12	5.79	24.073	0	0	7	0	0	1
13	5.991	30.548	0	0	5	0	0	1
14	5.78	24.940	0	0	7	1	0	1
15	5.073	15.122	0	0	5	1	0	1
16	5.738	26.634	0	0	5	1	0	1
17	5.879	15.160	0	0	7	0	0	1
18	5.807	14.349	0	0	7	0	0	1
19	6.102	21.673	0	0	5	0	0	1
20	5.86	14.891	0	0	7	1	0	1
21	5.115	0.000	0	0	4	0	0	2
22	4.928	6.163	0	0	4	0	0	2
23	5.189	0.000	0	0	4	0	0	2
24	5.471	0.000	0	0	4	0	0	2
25	5.556	0.000	0	0	4	0	0	2
26	5.295	0.000	0	0	4	0	0	2
27	5.421	0.000	0	0	4	0	0	2
28	5.502	0.000	0	0	4	0	0	2
29	5.391	0.000	0	0	4	0	0	2
30	5.034	0.000	0	0	4	0	0	2
31	5.04	9.885	0	0	4	0	0	2
32	5.46	0.000	0	0	4	0	0	2
33	5.342	0.000	0	0	4	0	0	2
34	5.354	5.693	0	0	4	0	0	2
35	5.6	0.000	0	0	4	0	0	2
36	4.746	0.000	0	0	4	0	0	2
37	5.481	13.277	0	0	4	0	0	2
38	5.399	13.294	0	0	4	0	0	2
39	5.772	13.036	0	0	4	0	0	2
40	5.243	0.000	0	0	4	0	0	2
41	5.91	0.000	0	0	8	0	0	2
42	4.914	0.000	0	0	4	0	0	2
43	5.298	0.000	0	0	4	0	0	2
44	5.264	0.000	0	0	4	0	0	2
45	5.456	0.000	0	0	4	0	0	2
46	5.59	0.000	0	0	4	0	0	2
47	5.437	0.000	0	0	0	0	0	2
48	5.339	0.000	0	0	4	0	0	1
49	5.22	0.000	0	0	4	0	0	1
50	4.942	5.074	0	0	4	0	0	1
51	4.931	5.927	0	0	3	0	0	1
52	5.248	8.676	0	0	6	2	0	1
53	4.943	0.000	0	0	6	2	0	1
54	5.315	0.000	0	0	6	4	0	1
55	4.681	6.545	1	0	3	0	0	2
56	4.556	6.796	1	0	6	0	0	2
57	4.674	13.405	1	0	2	1	0	3

58	4.633	6.606	1	0	4	1	0	3
59	4.361	6.688	1	0	4	1	0	3
60	4.482	13.355	1	0	3	1	0	2
61	4.756	0.000	1	0	4	0	0	2
62	4.623	6.647	1	0	3	0	0	2
63	4.814	6.638	1	0	3	1	0	2
64	5.163	13.286	1	0	2	1	0	2
65	4.566	6.627	1	0	4	1	0	2
66	4.673	6.661	1	0	4	1	0	3
67	4.762	17.301	1	0	5	1	0	3
68	5.223	25.703	1	0	3	1	0	3
69	4.634	19.233	0	0	5	2	0	3
70	5.143	29.428	0	0	4	2	0	2
71	4.87	25.966	0	0	2	0	0	3
72	6.027	17.685	0	0	4	0	0	2
73	6.42	25.685	0	0	4	0	0	2
74	5.91	25.772	0	0	4	0	0	2
75	5.415	25.147	0	0	6	0	0	2
76	5.914	25.551	0	0	6	0	0	1
77	5.314	20.012	0	0	4	0	0	2
78	5.754	20.029	0	0	10	0	0	2
79	5.793	19.936	0	0	4	0	0	2
80	4.991	22.128	0	0	8	0	0	2
81	6.26	30.750	0	0	4	0	0	1
82	5.489	20.876	0	1	3	1	0	1
83	5.333	21.026	0	1	3	1	0	2
84	5.116	8.567	0	0	3	1	0	1
85	5.511	20.835	0	1	3	1	0	1
86	5.83	20.950	0	0	3	1	0	2
87	5.499	34.315	0	0	4	1	0	2
88	5.274	8.649	0	0	3	1	0	2
89	5.423	8.484	0	0	3	1	0	2
90	4.594	6.916	1	0	0	0	0	5
91	4.388	0.000	0	0	4	3	0	1
92	5.164	6.653	0	0	4	3	0	1
93	4.641	6.726	1	0	2	0	0	2
94	4.494	12.772	0	0	4	2	0	3
95	4.916	11.891	0	0	2	1	0	3
96	5.1	18.555	0	0	1	1	0	3
97	5.159	11.725	0	0	2	1	0	3
98	5.063	11.731	0	0	1	1	0	2
99	5.226	11.900	0	0	1	2	0	3
100	4.994	11.796	0	0	2	1	0	3
101	5.139	18.544	0	0	0	1	0	3
102	5.09	11.793	0	0	2	1	0	3
103	5.103	11.834	0	0	2	1	0	3
104	4.883	18.352	0	0	0	1	0	3
105	5.233	11.809	0	0	2	1	0	3
106	5.025	11.689	0	0	1	1	0	2
107	4.445	12.055	0	0	2	1	0	4
108	4.71	12.065	0	0	2	1	0	3

109	4.546	18.480	0	0	0	1	0	3
110	4.46	11.963	0	0	1	1	0	2
111	4.991	11.734	0	0	2	1	0	3
112	4.722	11.853	0	0	2	3	0	3
113	4.655	11.911	0	0	1	1	0	3
114	5.05	18.423	0	0	0	1	0	3
115	4.835	17.700	0	0	1	1	0	3
116	5.105	17.773	0	0	2	1	0	3
117	4.872	12.034	0	0	2	1	0	4
118	4.767	11.975	0	0	1	1	0	3
119	5.024	31.948	0	0	1	1	0	3
120	4.989	12.051	0	0	2	1	0	4
121	5.107	18.485	0	0	4	0	0	1
122	5.306	18.440	0	0	4	0	0	1
123	5.917	18.418	0	0	4	0	0	1
124	5.578	18.454	0	0	4	0	0	2
125	5.078	18.410	0	0	4	0	0	2
126	5.674	25.003	0	0	4	0	0	1
127	6.959	24.420	0	0	8	0	0	1
128	5.979	20.423	0	0	8	0	0	2
129	6.357	20.683	0	0	7	0	0	2
130	6.387	20.683	0	0	7	0	0	2
131	5.558	18.563	0	0	4	1	0	1
132	5.793	24.213	0	0	7	0	0	2
133	5.489	24.294	0	0	7	0	0	2
134	5.65	31.079	0	0	6	0	0	2
135	5.358	31.123	0	0	6	0	0	2
136	5.561	24.241	0	0	7	0	0	2
137	6.721	24.085	0	0	8	0	0	2
138	6.62	23.968	0	0	11	0	0	2
139	5.936	31.536	0	0	6	0	0	2
140	5.59	24.052	0	0	7	0	0	2
141	5.272	24.074	0	0	4	0	0	2
142	6.456	24.012	0	0	8	0	0	2
143	6.252	24.098	0	0	8	0	0	1
144	6.585	24.098	0	0	8	0	0	1
145	5.815	24.240	0	0	5	0	0	2
146	5.539	24.116	0	0	5	0	0	2
147	5.064	24.116	0	0	5	0	0	2
148	5.212	11.668	0	0	2	1	0	0
149	5.523	18.474	0	0	0	1	0	0
150	5.319	25.334	0	0	0	1	0	0
151	5.701	18.697	0	0	1	0	0	0
152	5.243	11.589	0	0	0	0	0	0
153	5.544	18.411	0	0	0	0	0	0
154	7.097	24.698	0	0	9	1	0	1
155	6.108	12.043	0	0	4	0	0	0
156	4.066	11.705	0	0	2	0	0	2
157	5.229	32.557	0	0	7	1	0	1
158	6.367	25.736	0	0	8	2	0	1
159	5.157	27.937	0	0	6	1	0	0

160	4.658	6.655	0	0	6	0	0	1
161	5.147	27.512	0	0	1	0	0	0
162	5.305	15.290	0	0	1	0	0	0
163	5.092	15.194	0	0	1	0	0	0
164	5.783	15.429	0	0	1	0	0	0
165	5.377	15.332	0	0	1	0	0	0
166	5.09	15.209	0	0	1	0	0	0
167	5.498	15.250	0	0	1	0	0	0
168	5.313	15.382	0	0	1	0	0	0
169	5.035	15.347	0	0	1	0	0	0
170	7.301	5.869	0	1	0	0	2	0
171	7	5.913	0	1	0	0	2	0
172	7.398	5.983	0	1	0	0	2	0
173	6.921	5.953	0	1	0	0	2	0
174	7.155	5.877	0	1	0	0	2	0
175	7.155	5.903	0	1	0	0	2	0
176	7	5.837	0	1	0	0	2	0
177	7.155	5.890	0	1	0	0	2	0
178	7.155	5.799	0	1	0	0	2	0
179	7.222	12.607	0	1	0	0	2	0
180	7.301	12.610	0	1	0	0	2	0
181	7.301	5.824	0	1	0	0	2	0
182	7.222	5.928	0	1	0	0	2	0
183	7.301	5.874	0	1	0	0	1	0
184	7	18.773	0	1	0	0	2	0
185	7.301	5.855	0	1	0	0	2	0
186	7.097	5.954	0	1	0	0	2	0
187	7.155	5.919	0	1	0	2	2	0
188	7.097	5.839	0	1	0	2	2	0
189	6.959	5.912	0	0	0	0	2	0
190	6.585	6.092	0	0	0	0	2	0
191	6.638	5.898	0	0	0	0	2	0
192	6.886	5.920	0	0	0	0	2	0
193	6.796	5.903	0	0	0	0	2	0
194	4.648	6.280	0	0	2	0	0	2
195	4.831	6.240	0	0	2	1	0	2
196	4.776	6.261	0	0	2	0	0	2
197	4.429	6.271	0	0	0	0	0	2
198	5.12	6.169	0	0	2	0	0	2
199	5.229	6.223	0	0	2	1	0	2
200	5.026	6.152	0	0	2	0	0	2
201	5.042	6.328	0	0	2	0	0	2
202	5.303	6.285	0	0	2	1	0	2
203	4.645	6.176	0	0	2	0	0	2
204	5.051	6.272	0	0	2	1	0	2
205	5.554	6.361	0	0	2	0	0	2
206	4.957	6.259	0	0	2	1	0	2
207	5.194	6.256	0	0	2	0	0	2
208	4.844	6.190	0	0	2	1	0	2
209	4.575	12.857	0	0	2	0	0	2
210	5.324	12.900	0	0	2	1	0	2

211	5.228	6.255	0	0	2	1	0	2
212	5.301	6.216	0	0	2	0	0	2
213	5.682	6.262	0	0	2	1	0	2
214	5.745	6.257	0	0	2	0	0	2
215	5.412	6.255	0	0	2	0	0	2
216	5.353	6.302	0	0	2	1	0	2
217	5.205	13.092	0	0	2	0	0	2
218	4.485	12.858	0	0	2	1	0	2
219	5.143	22.342	1	0	2	1	0	1

Table S4: The SMILES notation for all two hundred and nineteen MDA-MB-231 cell anti-proliferative leads along with their reported IC_{50} and pIC_{50} values.

No	SMILES	IC_{50} (μM)	pIC_{50}
1	C1=C[C@@H]([C@]23[C@](C1=O)([C@@H]1[C@@H](C[C@H]2O3)[C@@]2([C@](CC1)(C(=C[C@@H]2OC(=O)C)[C@@H]([C@@H]1OC(=O)C(=C(C1)C)C)C)O)C)OC(=O)CCCC(=O)Nc1scnn1	2.17	5.664
2	C1=C[C@@H]([C@]23[C@](C1=O)([C@@H]1[C@@H](C[C@H]2O3)[C@@]2([C@](CC1)(C(=C[C@@H]2OC(=O)C)[C@@H]([C@@H]1OC(=O)C(=C(C1)C)C)C)O)C)OC(=O)CCCC(=O)Nc1sccn1	1.00	6.000
3	C1=C[C@@H]([C@]23[C@](C1=O)([C@@H]1[C@@H](C[C@H]2O3)[C@@]2([C@](CC1)(C(=C[C@@H]2OC(=O)C)[C@@H]([C@@H]1OC(=O)C(=C(C1)C)C)C)O)C)OC(=O)CCCC(=O)Nc1sc2c(n1)cccc2	0.67	6.174
4	C1=C[C@@H]([C@]23[C@](C1=O)([C@@H]1[C@@H](C[C@H]2O3)[C@@]2([C@](CC1)(C(=C[C@@H]2OC(=O)C)[C@@H]([C@@H]1OC(=O)C(=C(C1)C)C)C)O)C)OC(=O)CCCC(=O)Nc1ccc(cc1)F	0.49	6.31
5	C1=C[C@@H]([C@]23[C@](C1=O)([C@@H]1[C@@H](C[C@H]2O3)[C@@]2([C@](CC1)(C(=C[C@@H]2OC(=O)C)[C@@H]([C@@H]1OC(=O)C(=C(C1)C)C)C)O)C)OC(=O)CCCC(=O)Nc1ccc(cc1)N(C)C	1.07	5.971
6	C1=C[C@@H]([C@]23[C@](C1=O)([C@@H]1[C@@H](C[C@H]2O3)[C@@]2([C@](CC1)(C(=C[C@@H]2OC(=O)C)[C@@H]([C@@H]1OC(=O)C(=C(C1)C)C)C)O)C)OC(=O)CCCC(=O)Nc1ccc(cc1)OC	0.44	6.357
7	C1=C[C@@H]([C@]23[C@](C1=O)([C@@H]1[C@@H](C[C@H]2O3)[C@@]2([C@](CC1)(C(=C[C@@H]2OC(=O)C)[C@@H]([C@@H]1OC(=O)C(=C(C1)C)C)C)O)C)OC(=O)CCCC(=O)Nc1cc(c(cc1)N1CCOCC1)F	0.32	6.495
8	C1=C[C@@H]([C@]23[C@](C1=O)([C@@H]1[C@@H](C[C@H]2O3)[C@@]2([C@](CC1)(C(=C[C@@H]2OC(=O)C)[C@@H]([C@@H]1OC(=O)C(=C(C1)C)C)C)O)C)OC(=O)CCC(=O)Nc1scnn1	8.07	5.093
9	C1=C[C@@H]([C@]23[C@](C1=O)([C@@H]1[C@@H](C[C@H]2O3)[C@@]2([C@](CC1)(C(=C[C@@H]2OC(=O)C)[C@@H]([C@@H]1OC(=O)C(=C(C1)C)C)C)O)C)OC(=O)CCC(=O)Nc1sccn1	2.06	5.686
10	C1=C[C@@H]([C@]23[C@](C1=O)([C@@H]1[C@@H](C[C@H]2O3)[C@@]2([C@](CC1)(C(=C[C@@H]2OC(=O)C)[C@@H]([C@@H]1OC(=O)C(=C(C1)C)C)C)O)C)OC(=O)CCC(=O)Nc1sc2c(n1)cccc2	3.5	5.456
11	C1=C[C@@H]([C@]23[C@](C1=O)([C@@H]1[C@@H](C[C@H]2O3)[C@@]2([C@](CC1)(C(=C[C@@H]2OC(=O)C)[C@@H]([C@@H]1OC(=O)C(=C(C1)C)C)C)O)C)OC(=O)CCC(=O)Nc1ccc(cc1)F	1.87	5.728
12	C1=C[C@@H]([C@]23[C@](C1=O)([C@@H]1[C@@H](C[C@H]2O3)[C@@]2([C@](CC1)(C(=C[C@@H]2OC(=O)C)[C@@H]([C@@H]1OC(=O)C(=C(C1)C)C)C)O)C)OC(=O)CCC(=O)Nc1ccc(cc1)N(C)C	1.62	5.79
13	C1=C[C@@H]([C@]23[C@](C1=O)([C@@H]1[C@@H](C[C@H]2O3)[C@@]2([C@](CC1)(C(=C[C@@H]2OC(=O)C)[C@@H]([C@@H]1OC(=O)C(=C(C1)C)C)C)O)C)OC(=O)CCC(=O)Nc1ccc(cc1)OC	1.02	5.991
14	C1=C[C@@H]([C@]23[C@](C1=O)([C@@H]1[C@@H](C[C@H]2O3)[C@@]2([C@](CC1)(C(=C[C@@H]2OC(=O)C)[C@@H]([C@@H]1OC(=O)C(=C(C1)C)C)C)O)C)OC(=O)CCC(=O)Nc1cc(c(cc1)N1CCOCC1)F	1.66	5.78
15	C1=C[C@@H]([C@]23[C@](C1=O)([C@@H]1[C@@H](C[C@H]2O3)[C@@]2([C@](CC1)(C(=C[C@@H]2OC(=O)C)[C@@H]([C@@H]1OC(=O)C(=C(C1)C)C)C)O)C)OC(=O)/C=C\C(=O)Nc1scnn1	8.45	5.073
16	C1=C[C@@H]([C@]23[C@](C1=O)([C@@H]1[C@@H](C[C@H]2O3)[C@@]2([C@](CC1)(C(=C[C@@H]2OC(=O)C)[C@@H]([C@@H]1OC(=O)C(=C(C1)C)C)C)O)C)OC(=O)/C=C\C(=O)Nc1sccn1	1.83	5.738
17	C1=C[C@@H]([C@]23[C@](C1=O)([C@@H]1[C@@H](C[C@H]2O3)[C@@]2([C@](CC1)(C(=C[C@@H]2OC(=O)C)[C@@H]([C@@H]1OC(=O)C(=C(C1)C)C)C)O)C)OC(=O)/C=C\C(=O)Nc1ccc(cc1)F	1.32	5.879
18	C1=C[C@@H]([C@]23[C@](C1=O)([C@@H]1[C@@H](C[C@H]2O3)[C@@]2([C@](CC1)(C(=C[C@@H]2OC(=O)C)[C@@H]([C@@H]1OC(=O)C(=C(C1)C)C)C)O)C)OC(=O)/C=C\C(=O)Nc1ccc(cc1)N(C)C	1.56	5.807
19	C1=C[C@@H]([C@]23[C@](C1=O)([C@@H]1[C@@H](C[C@H]2O3)[C@@]2([C@](CC1)(C(=C[C@@H]2OC(=O)C)[C@@H]([C@@H]1OC(=O)C(=C(C1)C)C)C)O)C)OC(=O)/C=C\C(=O)Nc1ccc(cc1)OC	0.79	6.102
20	C1=C[C@@H]([C@]23[C@](C1=O)([C@@H]1[C@@H](C[C@H]2O3)[C@@]2([C@](CC1)(C(=C[C@@H]2OC(=O)C)[C@@H]([C@@H]1OC(=O)C(=C(C1)C)C)C)O)C)OC(=O)/C=C\C(=O)Nc1cc(c(cc1)N1CCOCC1)F	1.38	5.86
21	FC1=CC=C(C=C1)C(CCCCNC(=O)NC1=CC=C(C=C1)Cl)C1=CC=C(C=C1)F	7.68	5.115
22	FC1=CC=C(C=C1)C(CCCCNC(=O)NC1=CC=C(C=C1)I)C1=CC=C(C=C1)F	11.79	4.928
23	FC1=CC=C(C=C1)C(CCCCNC(=O)NC1=CC=C(C=C1)[N+](=O)[O-])C1=CC=C(C=C1)F	6.47	5.189
24	FC1=CC=C(C=C1)C(CCCCNC(=O)NC1=CC(=CC=C1)C(F)(F)F)C1=CC=C(C=C1)F	3.38	5.471
25	FC1=CC=C(C=C1)C(CCCCNC(=O)NC1=CC=C(C=C1)C(F)(F)F)C1=CC=C(C=C1)F	2.78	5.556
26	FC1=CC=C(C=C1)C(CCCCNC(=O)NC1=CC(=CC=C1)Cl)C1=CC=C(C=C1)F	5.07	5.295
27	FC1=CC=C(C=C1)C(CCCCNC(=O)NC1=CC(=CC=C1)[N+](=O)[O-])C1=CC=C(C=C1)F	3.79	5.421
28	FC1=CC=C(C=C1)C(CCCCNC(=O)NC1=CC(=C(C=C1)Cl)C(F)(F)F)C1=CC=C(C=C1)F	3.15	5.502

29	FC1=CC=C(C=C1)C(CCCCNC(=O)NC1=CC(=C(C=C1)Cl)[N+](=O)[O-])C1=CC=C(C=C1)F	4.06	5.391
30	NC=1C=C(C=CC1Cl)NC(=O)NCCCCC(C1=CC=C(C=C1)F)C1=CC=C(C=C1)F	9.24	5.034
31	FC1=CC=C(C=C1)C(CCCCNC(NC=1C=CC(=C(C1)NC(C)=O)Cl)=O)C1=CC=C(C=C1)F	9.12	5.04
32	FC1=CC=C(C=C1)C(CCCCNC(=O)NC1=CC(=C(C=C1)[N+](=O)[O-])Cl)C1=CC=C(C=C1)F	3.47	5.46
33	FC1=CC=C(C=C1)C(CCCCNC(=O)NC1=CC(=C(C=C1)[N+](=O)[O-])C(F)(F)F)C1=CC=C(C=C1)F	4.55	5.342
34	FC1=CC=C(C=C1)C(CCCCNC(NC1=CC(=C(C(=O)OC)C=C1)C(F)(F)F)=O)C1=CC=C(C=C1)F	4.43	5.354
35	ClC1=C(C=C(C=C1)NC(=O)NCCCCC(C1=CC=C(C=C1)C)C1=CC=C(C=C1)C)C(F)(F)F	2.51	5.6
36	ClC1=CC=C(C=C1)NC(=O)NCCCCC(C1=CC=C(C=C1)C)C1=CC=C(C=C1)C	17.93	4.746
37	COC1=CC=C(C=C1)C(CCCCNC(=O)NC1=CC(=C(C=C1)Cl)C(F)(F)F)C1=CC=C(C=C1)OC	3.3	5.481
38	COC1=CC=C(C=C1)C(CCCCNC(=O)NC1=CC(=C(C=C1)Cl)[N+](=O)[O-])C1=CC=C(C=C1)OC	3.99	5.399
39	FC(OC1=CC=C(C=C1)C(CCCCNC(=O)NC1=CC(=C(C=C1)Cl)C(F)(F)F)C1=CC=C(C=C1)OC(F)(F)F)(F)F	1.69	5.772
40	ClC1=C(C=C(C=C1)NC(=O)NCCCCC(C1=CC=CC=C1)C1=CC=CC=C1)C(F)(F)F	5.72	5.243
41	[N+](=O)([O-])C1=CC=C(C=C1)C(CCCCNC(=O)NC1=CC(=C(C=C1)Cl)C(F)(F)F)C1=CC=C(C=C1)[N+](=O)[O-]	1.23	5.91
42	NC1=CC=C(C=C1)C(CCCCNC(=O)NC1=CC(=C(C=C1)Cl)C(F)(F)F)C1=CC=C(C=C1)N	12.2	4.914
43	FC1=CC=C(C=C1)N(CCCCNC(=O)NC1=CC(=C(C=C1)Cl)C(F)(F)F)C1=CC=C(C=C1)F	5.04	5.298
44	ClC1=C(C=C(C=C1)NC(=O)NCCCCCC1=CC=C(C=C1)F)C(F)(F)F	5.44	5.264
45	FC1=CC=C(C=C1)C(CCCNC(=O)NC1=CC(=C(C=C1)Cl)C(F)(F)F)C1=CC=C(C=C1)F	3.5	5.456
46	FC1=CC=C(C=C1)C(CCCCCNC(=O)NC1=CC(=C(C=C1)Cl)C(F)(F)F)C1=CC=C(C=C1)F	2.57	5.59
47	FC1=CC=C(C=C1)C(CCCCNC(=S)NC1=CC(=C(C=C1)Cl)C(F)(F)F)C1=CC=C(C=C1)F	3.66	5.437
48	ClC1=C(C=C(C=C1)NC(OCCCCC(C1=CC=C(C=C1)F)C1=CC=C(C=C1)F)=O)C(F)(F)F	4.58	5.339
49	FC1=CC=C(C=C1)C(CCCCN(C(=O)NC1=CC(=C(C=C1)Cl)C(F)(F)F)C)C1=CC=C(C=C1)F	6.02	5.22
50	ClC1=C(C=C(C=C1)NC(CCCCC(C1=CC=C(C=C1)F)C1=CC=C(C=C1)F)=O)C(F)(F)F	11.44	4.942
51	FC1=CC=C(C=C1)C(CCCCNC(C1=CC(=C(C=C1)Cl)C(F)(F)F)=O)C1=CC=C(C=C1)F	11.71	4.931
52	FC1=CC=C(C=C1)C(=C1CCN(CC1)C(=O)NC1=CC(=C(C=C1)Cl)C(F)(F)F)C1=CC=C(C=C1)F	5.65	5.248
53	FC1=CC=C(C=C1)C(C1CCN(CC1)C(=O)NC1=CC(=C(C=C1)Cl)C(F)(F)F)C1=CC=C(C=C1)F	11.41	4.943
54	FC1=CC=C(C=C1)C(CC1CCN(CC1)C(=O)NC1=CC(=C(C=C1)Cl)C(F)(F)F)C1=CC=C(C=C1)F	4.84	5.315
55	FC1=CC=C(C=C1)NC1=NC(=NC(=C1)C(F)(F)F)NC1=CC=C(C=C1)NS(=O)(=O)C1=C(C=CC=C1)OC(F)(F)F	20.837	4.681
56	ClC1=C(C(=CC(=C1)C)OC)S(=O)(=O)NC1=CC=C(C=C1)NC1=NC(=CC(=N1)NC1=CC=C(C=C1)F)C(F)(F)F	27.783	4.556
57	COC1=CC=C(C=C1)S(=O)(=O)NC1=CC=C(C=C1)NC1=NC(=CC(=N1)NC1=CC=C(C=C1)OC)C(F)(F)F	21.186	4.674
58	COC1=CC=C(C=C1)NC1=NC(=NC(=C1)C(F)(F)F)NC1=CC=C(C=C1)NS(=O)(=O)C1=CC=C(C=C1)C(F)(F)F	23.293	4.633
59	ClC1=C(C=CC=C1)S(=O)(=O)NC1=CC=C(C=C1)NC1=NC(=CC(=N1)NC1=CC=C(C=C1)OC)C(F)(F)F	43.568	4.361
60	COC1=CC=C(C=C1)NC1=NC(=NC(=C1)C(F)(F)F)NC1=CC=C(C=C1)NS(=O)(=O)C1=C(C=CC=C1)OC(F)(F)F	32.946	4.482
61	FC1=CC=C(C=C1)NC1=NC(=NC(=C1)C)NC1=CC=C(C=C1)NS(=O)(=O)C1=CC=C(C=C1)C(F)(F)F	17.547	4.756
62	FC1=CC=C(C=C1)NC1=NC(=NC(=C1)C)NC1=CC=C(C=C1)NS(=O)(=O)C1=C(C=CC=C1)OC(F)(F)F	23.816	4.623
63	ClC=1C=C(C=CC1)S(=O)(=O)NC1=CC=C(C=C1)NC1=NC(=CC(=N1)NC1=CC=C(C=C1)OC)C	15.337	4.814
64	COC1=CC=C(C=C1)S(=O)(=O)NC1=CC=C(C=C1)NC1=NC=CC(=N1)NC1=CC=C(C=C1)OC	6.867	5.163
65	COC1=CC=C(C=C1)NC1=NC(=NC=C1)NC1=CC=C(C=C1)NS(=O)(=O)C1=CC=C(C=C1)C(F)(F)F	27.194	4.566
66	COC1=CC=C(C=C1)NC1=NC(=NC=C1)NC1=CC=C(C=C1)NS(=O)(=O)C1=CC=C(C=C1)C	21.229	4.673
67	COC1=CC(NC2=CC=NC(NC3=CC=C(NS(=O)(=O)C4=CC=C(C)C=C4)C=C3)=N2)=CC(OC)=C1OC	17.304	4.762
68	COC1=CC=C(C=C1)S(=O)(=O)NC1=CC=C(C=C1)NC1=NC=CC(=N1)NC1=CC(=C(C(=C1)OC)OC)OC	5.986	5.223
69	COC1=CC=C(C(=O)NC2=CC=C(C=C2)NC2=NC=CC(=N2)NC2=C(C=C(C=C2)OC)C)C=C1	23.251	4.634
70	COC1=C(C(=O)NC2=CC=C(C=C2)NC2=NC(=CC(=N2)C)NC2=CC(=C(C(=C2)OC)OC)OC)C=CC=C1	7.198	5.143
71	CN1C=NC2=CC=C(C=C2C1=O)C=1C=CC2=C(NC(=N2)NC(CC)=O)C1	13.5	4.87
72	CN1C(=NC2=CC=C(C=C2C1=O)C=1C=CC2=C(NC(=N2)NC(CC)=O)C1)CN1CCOCC1	0.94	6.027
73	O1CCN(CC1)CCN1C=NC2=CC=C(C=C2C1=O)C=1C=CC2=C(NC(=N2)NC(CC)=O)C1	0.38	6.42
74	O=C1N(C=NC2=CC=C(C=C12)C=1C=CC2=C(NC(=N2)NC(CC)=O)C1)CCN1CCCCC1	1.23	5.91
75	O1CCN(CC1)CCN1C=NC2=CC=C(C=C2C1=O)C=1C=CC2=C(NC(=N2)NC(CCCC)=O)C1	3.85	5.415
76	O1CCN(CC1)CCCN1C=NC2=CC=C(C=C2C1=O)C=1C=CC2=C(NC(=N2)NC(CC)=O)C1	1.22	5.914
77	CN(C(=O)NC1=NC2=C(N1)C=C(C=C2)C=2C=C1C(N(C=NC1=CC2)CCN2CCOCC2)=O)C	4.85	5.314

78	C(C)N(C(=O)NC1=NC2=C(N1)C=C(C=C2)C=2C=C1C(N(C=NC1=CC2)CCN2CCOCC2)=O)CC	1.76	5.754
79	O1CCN(CC1)CCN1C=NC2=CC=C(C=C2C1=O)C=1C=CC2=C(NC(=N2)NC(=O)C2CC2)C1	1.61	5.793
80	O1CCN(CC1)CCN1C=NC2=CC=C(C=C2C1=O)C=1C=CC2=C(NC(=N2)NC(=O)C2CCCCC2)C1	10.2	4.991
81	N1C=C(C2=CC=CC=C12)CCN1C=NC2=CC=C(C=C2C1=O)C=1C=CC2=C(NC(=N2)NC(CC)=O)C1	0.55	6.26
82	NC1=C(C=CC=C1)NC(C1=CC=C(C=C1)CN1C2=NC(=NC(=C2N=C1)NC1=CC(=C(C=C1)F)Cl)Cl)=O	3.24	5.489
83	NC1=C(C=CC=C1)NC(C1=CC=C(C=C1)CN1C2=NC(=NC(=C2N=C1)NC1=NNC=C1C)Cl)=O	4.64	5.333
84	NC1=C(C=CC=C1)NC(C1=CC=C(C=C1)CN1C=CC2=C1N=C(N=C2Cl)Cl)=O	7.65	5.116
85	NC1=NC2=C(N=CN2CC2=CC=C(C=C2)C(=O)NC2=C(N)C=CC=C2)C(Cl)=N1	3.08	5.511
86	NC1=NC(=C2N=CN(C2=N1)CC1=CC=C(C(=O)NC2=C(C=CC=C2)N)C=C1)NC1=CC(=C(C=C1)F)Cl	1.48	5.83
87	NC1=NC(=C2N=CN(C2=N1)CC1=CC=C(C(=O)NC2=C(C=CC=C2)N)C=C1)NC1=CC(=CC(=C1)OC)OC	3.17	5.499
88	NC=1N=C(C2=C(N1)N(C=C2)CC2=CC=C(C(=O)NC1=C(C=CC=C1)N)C=C2)NC2=CC(=C(C=C2)F)Cl	5.32	5.274
89	NC=1N=C(C2=C(N1)N(C=C2)CC2=CC=C(C(=O)NC1=C(C=CC(=C1)F)N)C=C2)Cl	3.78	5.423
90	FC=1C(NC(N(C1)CC(=O)NC1=C(C=CC(=C1)S(N)(=O)=O)O)=O)=O	25.46	4.594
91	ClC1=CC=C(CN(C(CN2C(NC(C(=C2)F)=O)=O)=O)CCC2=CC=C(C=C2)S(N)(=O)=O)C=C1	40.92	4.388
92	FC=1C(NC(N(C1)CC(=O)N(CCC1=CC=C(C=C1)S(N)(=O)=O)CC1=CC=C(C=C1)OC)=O)=O	6.86	5.164
93	FC=1C(NC(N(C1)CC=1N=NN(C1)C1=CC=C(C=C1)S(=O)(=O)N)=O)=O	22.85	4.641
94	FC=1C(NC(N(C1)CC(=O)NC1=CC=C2C(=CC(OC2=C1)=O)C)=O)=O	32.04	4.494
95	N1C=C(C2=CC=CC=C12)SC1=NC(=NC(=C1)N1CCOCC1)NC(=O)NC1=CC=CC=C1	12.14	4.916
96	N1C=C(C2=CC=CC=C12)SC1=NC(=NC(=C1)N1CCOCC1)NC(=O)NC1=CC(=CC=C1)OC	7.94	5.1
97	N1C=C(C2=CC=CC=C12)SC1=NC(=NC(=C1)N1CCOCC1)NC(=O)NC1=CC(=CC=C1)Cl	6.93	5.159
98	N1C=C(C2=CC=CC=C12)SC1=NC(=NC(=C1)N1CCOCC1)NC(=O)NC1=C(C=C(C=C1)Cl)C	8.65	5.063
99	N1C=C(C2=CC=CC=C12)SC1=NC(=NC(=C1)N1CCCCC1)NC(=O)NC1=C(C=CC(=C1)C)C	5.94	5.226
100	N1C=C(C2=CC=CC=C12)SC1=NC(=NC(=C1)N1CCCCC1)NC(=O)NC1=CC=C(C=C1)Cl	10.13	4.994
101	N1C=C(C2=CC=CC=C12)SC1=NC(=NC(=C1)N1CCCCC1)NC(=O)NC1=CC=C(C=C1)OC	7.26	5.139
102	N1C=C(C2=CC=CC=C12)SC1=NC(=NC(=C1)N1CCCCC1)NC(=O)NC1=CC=CC=C1	8.13	5.09
103	N1C=C(C2=CC=CC=C12)SC1=NC(=NC(=C1)N1CCCC1)NC(=O)NC1=CC=CC=C1	7.89	5.103
104	N1C=C(C2=CC=CC=C12)SC1=NC(=NC(=C1)N1CCCC1)NC(=O)NC1=CC=C(C=C1)OC	13.1	4.883
105	N1C=C(C2=CC=CC=C12)SC1=NC(=NC(=C1)N1CCCC1)NC(=O)NC1=CC=C(C=C1)Cl	5.85	5.233
106	N1C=C(C2=CC=CC=C12)SC1=NC(=NC(=C1)N1CCCC1)NC(=O)NC1=C(C=C(C=C1)Cl)C	9.44	5.025
107	N1C=C(C2=CC=CC=C12)SC1=NC(=NC(=C1)NC1=CC=CC=C1)NC(=O)NC1=CC=CC=C1	35.92	4.445
108	N1C=C(C2=CC=CC=C12)SC1=NC(=NC(=C1)N1CCN(CC1)C1=NC=CC=C1)NC(=O)NC1=CC=CC=C1	19.51	4.71
109	N1C=C(C2=CC=CC=C12)SC1=NC(=NC(=C1)N1CCN(CC1)C1=NC=CC=C1)NC(=O)NC1=CC=C(C=C1)O C	28.42	4.546
110	N1C=C(C2=CC=CC=C12)SC1=NC(=NC(=C1)N1CCN(CC1)C1=NC=CC=C1)NC(=O)NC1=C(C=C(C=C1)C l)C	34.7	4.46
111	N1C=C(C2=CC=CC=C12)SC1=NC(=NC(=C1)N1CCN(CC1)CC1=CC=CC=C1)NC(=O)NC1=CC=CC=C1	10.22	4.991
112	N1C=C(C2=CC=CC=C12)SC1=NC(=NC(=C1)N1CCN(CC1)CC1=CC=CC=C1)NC(=O)NC1=CC(=CC(=C1) C)C	18.96	4.722
113	N1C=C(C2=CC=CC=C12)SC1=NC(=NC(=C1)N1CCN(CC1)CC1=CC=CC=C1)NC(=O)NC1=C(C=C(C=C1) Cl)C	22.13	4.655
114	N1C=C(C2=CC=CC=C12)SC1=NC(=NC(=C1)N1CCN(CC1)CC1=CC=CC=C1)NC(=O)NC1=CC=C(C=C1) OC	8.92	5.05
115	N1C=C(C2=CC=CC=C12)SC1=NC(=NC(=C1)NC1CC1)NC(=O)NC1=C(C=C(C=C1)Cl)C	14.61	4.835
116	N1C=C(C2=CC=CC=C12)SC1=NC(=NC(=C1)NC1CC1)NC(=O)NC1=CC=CC=C1	7.86	5.105
117	N1C=C(C2=CC=CC=C12)SC1=NC(=NC(=C1)NC1=CC=CC=C1)NC(=O)NC1=CC=C(C=C1)C	13.42	4.872
118	N1C=C(C2=CC=CC=C12)SC1=NC(=NC(=C1)NC1=CC=CC=C1)NC(=O)NC1=C(C=C(C=C1)Cl)C	17.1	4.767
119	N1C=C(C2=CC=CC=C12)SC1=NC(=NC(=C1)NC1=CC(=CC(=C1)OC)OC)NC(=O)NC1=CC=C(C=C1)OC	9.46	5.024
120	N1C=C(C2=CC=CC=C12)SC1=NC(=NC(=C1)NC1=CC=C(C=C1)Cl)NC(=O)NC1=CC=CC=C1	10.26	4.989
121	COC1=C(C=CC=C1)C1=CSC2=C1N=C(N=C2)NC2=CC=C(CP(OC)(OC)=O)C=C2	7.81	5.107
122	COC1=C(C=CC=C1)C1=CSC2=C1N=C(N=C2)NC2=CC=C(CP(OCC)(OCC)=O)C=C2	4.94	5.306
123	COC1=C(C=CC=C1)C1=CSC2=C1N=C(N=C2)NC2=CC=C(CP(OCC)(=O)NC)C=C2	1.21	5.917
124	OC(C1=CC=C(C=C1)NC=1N=CC2=C(N1)C(=CS2)C2=C(C=CC=C2)OC)P(OCC)(OCC)=O	2.64	5.578
125	COC1=C(C=CC=C1)C1=CSC2=C1N=C(N=C2)NC2=CC=C(CP(OCC)(=O)N1CCOCC1)C=C2	8.35	5.078
126	COC1=C(C=CC=C1)C1=CSC2=C1N=C(N=C2)NC2=CC=C(C(=O)NC)C=C2	2.12	5.674

127	COC1=C(C=CC=C1)C1=CSC2=C1N=C(N=C2)NC2=CC=C(C(=O)NC1CCNCC1)C=C2	0.11	6.959
128	COC1=C(C=CC=C1)C1=CSC2=C1N=C(N=C2)NC2=CC=C(C=C2)NC(=O)C2CCNCC2	1.05	5.979
129	COC1=C(C=CC=C1)C1=CSC2=C1N=C(N=C2)NC2=CC=C(C=C2)NC(=O)[C@@H]2CNCCC2	0.44	6.357
130	COC1=C(C=CC=C1)C1=CSC2=C1N=C(N=C2)NC2=CC=C(C=C2)NC(=O)[C@H]2CNCCC2	0.41	6.387
131	NCC(=O)NC1=CC=C(C=C1)NC=1N=CC2=C(N1)C(=CS2)C2=C(C=CC=C2)OC	2.77	5.558
132	FC=1C=C(C(=O)NC2CCNCC2)C=CC1NC=1N=CC2=C(N1)C(=CS2)C2=C(C=CC=C2)OC	1.61	5.793
133	COC1=C(C=CC=C1)C1=CSC2=C1N=C(N=C2)NC2=C(C=C(C(=O)NC1CCNCC1)C=C2)C	3.24	5.489
134	COC=1C=C(C(=O)NC2CCNCC2)C=CC1NC=1N=CC2=C(N1)C(=CS2)C2=C(C=CC=C2)OC	2.24	5.65
135	C(C)OC=1C=C(C(=O)NC2CCNCC2)C=CC1NC=1N=CC2=C(N1)C(=CS2)C2=C(C=CC=C2)OC	4.39	5.358
136	COC1=C(C=CC=C1)C1=CSC2=C1N=C(N=C2)NC2=C(C=C(C(=O)NC1CCNCC1)C=C2)C(F)(F)F	2.75	5.561
137	FC1=C(C(=O)NC2CCNCC2)C=CC(=C1)NC=1N=CC2=C(N1)C(=CS2)C2=C(C=CC=C2)OC	0.19	6.721
138	COC1=C(C=CC=C1)C1=CSC2=C1N=C(N=C2)NC2=CC(=C(C(=O)NC1CCNCC1)C=C2)C	0.24	6.62
139	FC1=C(C(=O)NC2CCNCC2)C=C(C(=C1)NC=1N=CC2=C(N1)C(=CS2)C2=C(C=CC=C2)OC)OC	1.16	5.936
140	FC1=C(C(=O)NC2CCNCC2)C=C(C(=C1)NC=1N=CC2=C(N1)C(=CS2)C2=C(C=CC=C2)OC)F	2.57	5.59
141	FC1=C(C(=O)NC2CCOCC2)C=CC(=C1)NC=1N=CC2=C(N1)C(=CS2)C2=C(C=CC=C2)OC	5.35	5.272
142	FC1=C(C(=O)NC2CCN(CC2)C)C=CC(=C1)NC=1N=CC2=C(N1)C(=CS2)C2=C(C=CC=C2)OC	0.35	6.456
143	FC1=C(C(=O)N[C@@H]2CNCC2)C=CC(=C1)NC=1N=CC2=C(N1)C(=CS2)C2=C(C=CC=C2)OC	0.56	6.252
144	FC1=C(C(=O)N[C@H]2CNCC2)C=CC(=C1)NC=1N=CC2=C(N1)C(=CS2)C2=C(C=CC=C2)OC	0.26	6.585
145	FC1=C(C(=O)NCC2CCNCC2)C=CC(=C1)NC=1N=CC2=C(N1)C(=CS2)C2=C(C=CC=C2)OC	1.53	5.815
146	FC1=C(C(=O)NC[C@H]2CNCCC2)C=CC(=C1)NC=1N=CC2=C(N1)C(=CS2)C2=C(C=CC=C2)OC	2.89	5.539
147	FC1=C(C(=O)NC[C@@H]2CNCCC2)C=CC(=C1)NC=1N=CC2=C(N1)C(=CS2)C2=C(C=CC=C2)OC	8.62	5.064
148	CSC1=CC=C(C=C1)C(\C=C\C1=CC=C(C=C1)N1CCOCC1)=O	6.14	5.212
149	COC=1C=C(C=CC1)C(\C=C\C1=CC=C(C=C1)N1CCCC1)=O	3	5.523
150	O1COC2=C1C=CC(=C2)C(\C=C\C2=CC=C(C=C2)N2CCCC2)=O	4.8	5.319
151	ClCCN(C1=CC=C(C=C1)/C=C/C(=O)C1=C(C=CC=C1)OC)CCCl	1.99	5.701
152	ClCCN(C1=CC=C(C=C1)/C=C/C(=O)C1=CC=C(C=C1)S(=O)(=O)C)CCCl	5.71	5.243
153	ClCCN(C1=CC=C(C=C1)/C=C/C(=O)C1=CC(=CC=C1)OC)CCCl	2.86	5.544
154	CNC(=O)C1=CC=C(\C=C\C(=O)NCC(=O)N(C)C2=C(Cl)C(COC3=CC=CC4=C3N=C(C)C=C4OCC3=NC=C C=C3)=C(Cl)C=C2)C=C1	0.08	7.097
155	CN(C(=O)CN1C(=O)C2=C(C=CC=C2)C1=O)C1=C(Cl)C(CO)=C(Cl)C=C1	0.78	6.108
156	CNC(=O)C1=CC=C(\C=C\C(O)=O)C=C1	86	4.066
157	COC1=C(O)C=CC(\C=C\C(=O)NCC(=O)N(C)C2=C(Cl)C(COC3=CC=CC4=C3N=C(C)C=C4OCC3=NC=C C=C3)=C(Cl)C=C2)=C1	5.9	5.229
158	CNC(=O)C1=CC=C(\C=C\C(=O)NCC2=CN(N=N2)C2=C(Cl)C(CNC3=CC=CC4=C3N=C(C)C=C4OCC3=N C=CC=C3)=C(Cl)C=C2)C=C1	0.43	6.367
159	CC1=NC2=C(C=CC=C2OCC2=CC=CC(OC3=CC=CC=C3)=C2)C(OCC2=NC=CC=C2)=C1	6.97	5.157
160	CC1=NC2=C(C=CC=C2N)C(OCC2=NC=CC=C2)=C1	22	4.658
161	C(C)(C)(C)N1N=C2C(=C1)OC1(CC2=O)CCN(CC1)C(=O)C1=CC(=NC2=CC=CC=C12)C1=CC(=C(C=C1) OC)OC	7.13	5.147
162	NC1=CC=C(C=C1)C1=NC2=CC=CC=C2C(=C1)C(=O)N1CCC2(CC(C3=NN(C=C3O2)C(C)(C)C)=O)CC1	4.96	5.305
163	C(C)(C)(C)N1N=C2C(=C1)OC1(CC2=O)CCN(CC1)C(=O)C1=CC(=NC2=CC=CC=C12)C1=NC=CC=C1	8.1	5.092
164	C(C)(C)(C)N1N=C2C(=C1)OC1(CC2=O)CCN(CC1)C(=O)C1=CC(=NC2=CC=CC=C12)C1=CC=NC=C1	1.65	5.783
165	C(C)(C)(C)N1N=C2C(=C1)OC1(CC2=O)CCN(CC1)C(=O)C1=CC(=NC2=CC=CC=C12)N1CCNCC1	4.2	5.377
166	C(C)(C)(C)N1N=C2C(=C1)OC1(CC2=O)CCN(CC1)C(=O)C1=CC(=NC2=CC=CC=C12)N1CCN(CC1)C	8.13	5.09
167	C(C)(C)(C)N1N=C2C(=C1)OC1(CC2=O)CCN(CC1)C(=O)C1=CC(=NC2=CC=CC=C12)N1CCOCC1	3.18	5.498
168	C(C)(C)(C)N1N=C2C(=C1)OC1(CC2=O)CCN(CC1)C(=O)C1=CC(=NC2=CC=CC=C12)C	4.86	5.313
169	C(C)(C)(C)N1N=C2C(=C1)OC1(CC2=O)CCN(CC1)C(=O)C1=CC(=NC2=CC=CC=C12)Cl	9.22	5.035
170	C1(=CC=CC2=CC=CC=C12)C1=NN(C(=N1)N1C(COC12C=CC(C=C2)=O)=O)C2=CC=CC=C2	0.05	7.301
171	CC1OC2(N(C1=O)C1=NC(=NN1C1=CC=CC=C1)C1=CC=CC3=CC=CC=C13)C=CC(C=C2)=O	0.10	7.000
172	C1(=CC=CC2=CC=CC=C12)C1=NN(C(=N1)N1C(COC12C=CC(C=C2)=O)=O)C2=CC=C(C=C2)C	0.04	7.398
173	C1(=CC=CC2=CC=CC=C12)C1=NN(C(=N1)N1C(COC12C=CC(C=C2)=O)=O)C2=C(C=CC=C2)C	0.12	6.921
174	C1=C(C=CC2=CC=CC=C12)C1=NN(C(=N1)N1C(COC12C=CC(C=C2)=O)=O)C2=CC=CC=C2	0.07	7.155
175	CC1OC2(N(C1=O)C1=NC(=NN1C1=CC=CC=C1)C1=CC3=CC=CC=C3C=C1)C=CC(C=C2)=O	0.07	7.155

176	CC1OC2(N(C1=O)C1=NC(=NN1C1=CC=C(C=C1)C)C1=CC3=CC=CC=C3C=C1)C=CC(C=C2)=O	0.10	7.000
177	ClC1=CC=C(C=C1)N1N=C(N=C1N1C(COC12C=CC(C=C2)=O)=O)C2=CC1=CC=CC=C1C=C2	0.07	7.155
178	C1=C(C=CC2=CC=CC=C12)C1=NN(C(=N1)N1C(COC12C=CC(C=C2)=O)=O)C2=CC=C(C=C2)C(F)(F)F	0.07	7.155
179	COC1=CC=C(C=C1)N1N=C(N=C1N1C(COC12C=CC(C=C2)=O)=O)C2=CC1=CC=CC=C1C=C2	0.06	7.222
180	COC1=CC=C(C=C1)N1N=C(N=C1N1C(C(OC12C=CC(C=C2)=O)C)=O)C2=CC1=CC=CC=C1C=C2	0.05	7.301
181	C1=C(C=CC2=CC=CC=C12)N1N=C(N=C1N1C(COC12C=CC(C=C2)=O)=O)C2=CC1=CC=CC=C1C=C2	0.05	7.301
182	C1=C(C=CC2=CC=CC=C12)N1N=C(N=C1N1C(C(OC12C=CC(C=C2)=O)C)=O)C2=CC1=CC=CC=C1C=C2	0.06	7.222
183	BrC1=C(C=CC2=CC=CC=C12)C1=NN(C(=N1)N1C(COC12C=CC(C=C2)=O)=O)C2=CC=CC=C2	0.05	7.301
184	O1C2=C(OCC1)C=C(C=C2)C2=NN(C(=N2)N2C(COC21C=CC(C=C1)=O)=O)C1=CC=CC=C1	0.10	7.000
185	C1(=CC=CC=C1)N1N=C(N=C1N1C(COC12C=CC(C=C2)=O)=O)C=2C=C1C=CC=NC1=CC2	0.05	7.301
186	FC1=CC=C(C2=CC=CC=C12)C1=NN(C(=N1)N1C(COC12C=CC(C=C2)=O)=O)C2=CC=CC=C2	0.08	7.097
187	C1=C(C=CC=2C3=CC=CC=C3CC12)C1=NN(C(=N1)N1C(COC12C=CC(C=C2)=O)=O)C2=CC=CC=C2	0.07	7.155
188	C1=C(C=CC=2C3=CC=CC=C3CC12)C1=NN(C(=N1)N1C(COC12C=CC(C=C2)=O)=O)C2=CC=C(C=C2)C(F)(F)F	0.08	7.097
189	C1(=CC=CC2=CC=CC=C12)C1=NN(C(=N1)N1CCOC12C=CC(C=C2)=O)C2=CC=CC=C2	0.11	6.959
190	C1(=CC=CC2=CC=CC=C12)C1=NN(C(=N1)N1CCOC12C=CC(C=C2)=O)C2=CC=C(C=C2)C	0.26	6.585
191	C1=C(C=CC2=CC=CC=C12)C1=NN(C(=N1)N1CCOC12C=CC(C=C2)=O)C2=CC=CC=C2	0.23	6.638
192	CC1OC2(N(C1)C1=NC(=NN1C1=CC=CC=C1)C1=CC3=CC=CC=C3C=C1)C=CC(C=C2)=O	0.13	6.886
193	C1=C(C=CC2=CC=CC=C12)C1=NN(C(=N1)N1CCOC12C=CC(C=C2)=O)C2=CC=C(C=C2)C	0.16	6.796
194	S1C(=NC2=C1C=CC=C2)NC2=NC(=NC=C2C)NC2=CC=C(C=C2)[N+](=O)[O-]	22.49	4.648
195	S1C(=NC2=C1C=CC=C2)NC2=NC(=NC=C2C)NC2=CC=C(C=C2)S(=O)(=O)C	14.76	4.831
196	S1C(=NC2=C1C=CC=C2)NC2=NC(=NC=C2C)NC2=CC=C(C=C2)S(=O)(=O)N	16.75	4.776
197	S1C(=NC2=C1C=CC=C2)NC2=NC(=NC=C2C)NC2=CC=C(C=C2)N2CCCCC2	37.23	4.429
198	FC1=CC2=C(N=C(S2)NC2=NC(=NC=C2C)NC2=CC=C(C=C2)S(=O)(=O)N)C=C1	7.58	5.12
199	ClC1=CC2=C(N=C(S2)NC2=NC(=NC=C2C)NC2=CC=C(C=C2)S(=O)(=O)C)C=C1	5.9	5.229
200	ClC1=CC2=C(N=C(S2)NC2=NC(=NC=C2C)NC2=CC=C(C=C2)S(=O)(=O)N)C=C1	9.42	5.026
201	CC=1C(=NC(=NC1)NC1=CC=C(C=C1)S(=O)(=O)N)NC=1SC2=C(N1)C=CC(=C2)C	9.08	5.042
202	S1C(=NC2=C1C=CC=C2)NC2=NC(=NC=C2F)NC2=CC=C(C=C2)S(=O)(=O)C	4.98	5.303
203	S1C(=NC2=C1C=CC=C2)NC2=NC(=NC=C2F)NC2=CC=C(C=C2)S(=O)(=O)N	22.63	4.645
204	FC=1C(=NC(=NC1)NC1=CC=C(C=C1)S(=O)(=O)C)NC=1SC2=C(N1)C=CC(=C2)F	8.9	5.051
205	ClC1=CC2=C(N=C(S2)NC2=NC(=NC=C2F)NC2=CC=C(C=C2)S(=O)(=O)N)C=C1	2.79	5.554
206	ClC1=CC2=C(N=C(S2)NC2=NC(=NC=C2F)NC2=CC=C(C=C2)S(=O)(=O)C)C=C1	11.05	4.957
207	FC=1C(=NC(=NC1)NC1=CC=C(C=C1)S(=O)(=O)N)NC=1SC2=C(N1)C=CC(=C2)C	6.39	5.194
208	FC=1C(=NC(=NC1)NC1=CC=C(C=C1)S(=O)(=O)C)NC=1SC2=C(N1)C=CC(=C2)C	14.33	4.844
209	FC=1C(=NC(=NC1)NC1=CC=C(C=C1)S(=O)(=O)N)NC=1SC2=C(N1)C=CC(=C2)OC	26.6	4.575
210	FC=1C(=NC(=NC1)NC1=CC=C(C=C1)S(=O)(=O)C)NC=1SC2=C(N1)C=CC(=C2)OC	4.74	5.324
211	S1C(=NC2=C1C=CC=C2)NC2=NC(=NC=C2)NC2=CC=C(C=C2)S(=O)(=O)C	5.91	5.228
212	S1C(=NC2=C1C=CC=C2)NC2=NC(=NC=C2)NC2=CC=C(C=C2)S(=O)(=O)N	5.00	5.301
213	FC1=CC2=C(N=C(S2)NC2=NC(=NC=C2)NC2=CC=C(C=C2)S(=O)(=O)C)C=C1	2.08	5.682
214	FC1=CC2=C(N=C(S2)NC2=NC(=NC=C2)NC2=CC=C(C=C2)S(=O)(=O)N)C=C1	1.80	5.745
215	CC1=CC2=C(N=C(S2)NC2=NC(=NC=C2)NC2=CC=C(C=C2)S(=O)(=O)N)C=C1	3.87	5.412
216	CS(=O)(=O)C1=CC=C(C=C1)NC1=NC=CC(=N1)NC=1SC2=C(N1)C=CC(=C2)C	4.44	5.353
217	COC1=CC2=C(N=C(S2)NC2=NC(=NC=C2)NC2=CC=C(C=C2)S(=O)(=O)N)C=C1	6.24	5.205
218	CS(=O)(=O)C1=CC=C(C=C1)NC1=NC=CC(=N1)NC=1SC2=C(N1)C=CC(=C2)OC	32.74	4.485
219	CC(C)N1C(C)=NC=C1C1=CC=NC(NC2=CC=C(C=C2)S(C)(=O)=O)=N1	7.20	5.143

➢ **Statistical parameters for used for validation of QSAR models**

$$R^2 = 1 - \frac{\sum(y_i - \hat{y}_i)^2}{\sum(y_i - \overline{y})^2}$$

Where, y_i are the observed values of the response, ȳ the corresponding average, ŷ are the calculated values

$$Q^2 = 1 - \frac{\sum(y_i - \hat{y}_i)^2}{\sum(y_i - \overline{y})^2}$$

Where, y_i are the observed values of the response, ȳ the corresponding average, ŷ are the values predicted for each object when it is not in the training set.

$$Q_{F1}^2 = 1 - \frac{\sum_{i=1}^{n_{EXT}}(y_i - \hat{y}_l)^2}{\sum_{i=1}^{n_{EXT}}(y_i - \overline{y}_{TR})^2}$$

Where, y_i are the observed values of the response, ȳ the corresponding average, ŷ are the calculated values

$$Q_{F2}^2 = 1 - \frac{\sum_{i=1}^{n_{EXT}}(y_i - \hat{y}_l)^2}{\sum_{i=1}^{n_{EXT}}(y_i - \overline{y}_{EXT})^2}$$

Where, y_i are the observed values of the response, ȳ the corresponding average, ŷ are the calculated values

$$Q_{F3}^2 = 1 - \frac{\left[\sum_{i=1}^{n_{EXT}}(y_i - \hat{y}_l)^2\right] / n_{EXT}}{\left[\sum_{i=1}^{n_{TR}}(y_i - \overline{y}_{TR})^2\right] / n_{TR}}$$

Where, y_i are the observed values of the response, ȳ the corresponding average, ŷ are the calculated values

$$CCC = \frac{2\sum_{i=1}^{n_{EXT}}(y_i - \overline{y})(\hat{y}_i - \overline{y})}{\sum_{i=1}^{n_{EXT}}(y_i - \overline{y})^2 + \sum_{i=1}^{n_{EXT}}(\hat{y}_i - \overline{\hat{y}})^2 + n_{EXT}(\overline{y} - \overline{\hat{y}})^2}$$

$$k = \frac{\sum_{i=1}^{n_{EXT}} y_i\hat{y}_i}{\sum_{i=1}^{n_{EXT}} \hat{y}_i^{\,2}}$$

$$k' = \frac{\sum_{i=1}^{n_{EXT}} y_i\hat{y}_i}{\sum_{i=1}^{n_{EXT}} y_i^{\,2}}$$

$$r_m^2 = r^2(1 - \sqrt{r^2 - r_0^{\,2}})$$

$$\overline{r_m^{\,2}} = \frac{(r_m^{\,2} + r'^{\,2}_m)}{2}$$

$$RMSE = \sqrt{\frac{\sum_{i=1}^{n_{EXT}} (y_i - \hat{y}_i)^2}{n_{EXT}}}$$

Where, y_i are the observed values of the response, $\bar{y}$ the corresponding average, $\hat{y}$ are the calculated values

$$MAE = \frac{\sum_{i=1}^{n_{EXT}} |y_i - \hat{y}_i|}{n_{EXT}}$$

- **Definitions of the Molecular Descriptors derived from PyDescriptor and appeared in QSAR Model 1.1 and 1.2.**

Sr. No.	Molecular Descriptor	Definition
1	**all_MSA3**	Molecular Surface Area of all atoms having partial charge in the range -0.099 to 0.000
2	**com_Splus_7A**	Number of positively charged Sulfur atoms within 7Å from center of mass of molecule
3	**fHringC4B**	Frequency of occurrence of ring carbons which are present exactly at 4 bonds from hydrogen atom
4	**fOH5B**	Frequency of occurrence of number of hydrogen atoms which are present exactly at 5 bonds from Oxygen atom
5	**fringNC8B**	Frequency of occurrence of number of carbon atoms which are present exactly at 8 bonds from ring Nitrogen atoms
6	**com_sp2N_2A**	Number of sp2-Nitrogen atoms within 2Å from center of mass of molecule
7	**com_don_6A**	Number of donor atoms within 6Å from center of mass of molecule

Table S5: Definitions of Molecular Descriptors in QSAR Models.

****
